# Translating gut microbiome research into therapies for type 1 diabetes

**DOI:** 10.1113/EP093273

**Published:** 2026-09-10

**Authors:** Shanti P. Kok, Max Nieuwdorp, Elena Rampanelli

**Affiliations:** ^1^ Department of Vascular Medicine, Amsterdam University Medical Centre University of Amsterdam Amsterdam The Netherlands; ^2^ Diabeter Centre Amsterdam (DCA) Amsterdam The Netherlands; ^3^ Department of Experimental Vascular Medicine, Amsterdam University Medical Centre University of Amsterdam Amsterdam The Netherlands; ^4^ Amsterdam Amsterdam institute for Immunology and Infectious diseases (AII) Amsterdam The Netherlands

**Keywords:** gut microbiome, microbial metabolites, microbiome‐based therapies, type 1 diabetes

## Abstract

Type 1 diabetes (T1D) is characterised by the loss of functional pancreatic β‐cells, for which lifelong insulin therapy remains the standard of care. Given that the gut microbiome can influence host health and that shifts in gut microbial profiles have been observed in T1D, growing interest has emerged in the role of the gut microbiome in T1D, particularly for its therapeutic potential. The current review aims to provide an overview of existing knowledge on the gut microbial metabolic pathways and microbiota‐derived metabolites that are dysregulated or altered in T1D. Subsequently, we address recent advances in gut microbiome‐based therapies, ranging from prebiotics to faecal microbiota transplantation (FMT) in both preclinical and clinical studies. Observational studies in T1D have demonstrated alterations in short‐chain fatty acid, secondary bile acid and tryptophan metabolism, as well as changes in the abundance of certain gut microbes and the expression of microbial genes involved in these metabolic pathways. Moreover, gut microbiome‐targeting strategies have been shown to improve certain glycaemic and immunological parameters in T1D as well as the gut microbial community composition. Nonetheless, findings are highly heterogeneous among studies, also depending on the characteristics of the population studied. While microbiome‐based interventions hold promise as a novel therapeutic approach for T1D, expanding the repertoire of gut microbiome‐based therapies and conducting more robust clinical trials are crucial steps to substantiate current findings and establish their efficacy before these interventions can be applied as adjunctive therapies in T1D.

## Abstract (highlights)

- **What is the topic of this review?**
  This review addresses the role of the gut microbiome in type 1 diabetes (T1D) and the potential of gut microbiome‐based therapies to prevent and treat T1D.
- **What advances does it highlight?**
  The gut microbiome structure and function are altered in T1D. Faecal microbiota transplantation studies support the microbiota impact on host immunity and β‐cell function. Various gut microbiome‐based therapies are being explored in preclinical and clinical studies as modulators of (auto)immunity, the gut microbiota and metabolic profiles. However, results vary and clinical effects on β‐cell function remain scarce in human T1D.

## 1 INTRODUCTION

Type 1 diabetes (T1D) is a T cell‐mediated autoimmune disease characterised by pancreatic β‐cell destruction, resulting in insulin deficiency (Henriques et al., 2025; Huang et al., 2024). Despite efforts to develop alternative interventions for T1D, insulin therapy remains the current gold standard of treatment (Pathak et al., 2019). Lifelong insulin therapy is essential for survival (Henriques et al., 2025) and for reducing the incidence of T1D‐related complications (International Diabetes Federation, 2025). Nevertheless, optimal glycaemic control is achieved by only a minority, with one study reporting optimal control in just 17% of youths and 21% of adults (Foster et al., 2019). Depending on age at diagnosis, T1D can reduce life expectancy by 7.6–17.7 years (Heald et al., 2020; Rawshani et al., 2018).

An estimated 9.15 million individuals were living with T1D in 2024 (International Diabetes Federation, 2025), and this number is projected  to rise to 14.7 million in 2040 (Ogle et al., 2025). Over time, a growing consensus has emerged that genetics alone cannot explain this rising incidence (Garcia‐Gutierrez et al., 2024). Notably, interest in the role of the gut microbiome in T1D development and pathogenesis has increased in the recent decades (Garcia‐Gutierrez et al., 2024; Huang et al., 2024).

The gut microbiome consists of a vast number of living microorganisms, along with their associated genomes, structural elements, metabolites and environmental conditions (Hou et al., 2022). Within this complex ecosystem, mostly gut bacteria have been the focus of research due to their profound effects on health and disease. They influence host health by generating specific metabolites, synthesising vitamins, inhibiting pathogen growth or colonialisation, and interacting with the host immune system.

Although gut microbiome alterations across various stages of T1D have been reviewed elsewhere (Blok et al., 2025), studies consistently report a decline in microbial diversity, an increased Bacteroidetes‐to‐Firmicutes ratio, and a higher abundance of pathogenic bacteria (Huang et al., 2024). To date, The Environmental Determinants of Diabetes in the Young (TEDDY) study remains the most prominent study exploring T1D‐associated gut microbiome alterations (Stewart et al., 2018; Vatanen et al., 2018). Notably, T1D development is associated with reduced abundance of 11 bacterial genera, including *Akkermansia*, *Lactococcus*, *Streptococcus* and unclassified *Ruminococcaceae*, whereas five other genera, including *Parabacteroides*, were significantly correlated with T1D onset (Stewart et al., 2018).

Notably, the gut microbiota possesses a huge metabolic capacity producing an array of metabolites that can act as signalling molecules in the host, thereby affecting host physiology. Building on early evidence of gut microbiome alterations in T1D, various studies are currently investigating potential targets for intervention and different gut microbiome‐based therapies, ranging from prebiotics to faecal microbiota transplantation (FMT). In this narrative review, we summarize and discuss the role of microbiota‐derived metabolites in T1D, with a particular focus on their therapeutic potential. We examine evidence from both preclinical models and clinical studies, highlighting how these metabolites influence disease pathogenesis and evaluating their potential as novel therapeutic interventions.

## 2 GUT MICROBIAL METABOLITES IN T1D

**FIGURE 1:**
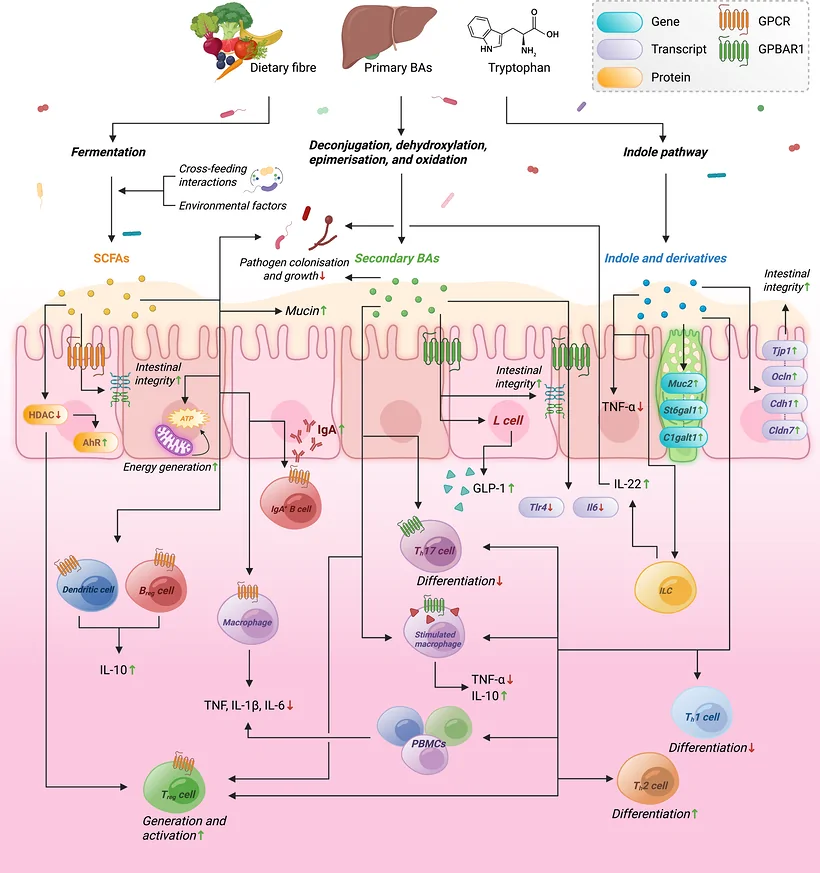
Graphical summary of the physiological effects of the various metabolites on mucosal immunity and intestinal integrity. In general, these metabolites, including SCFAs (yellow), secondary BAs (green), and tryptophan‐derived indoles and indole derivatives (blue), promote immune defence, intestinal integrity and a more tolerogenic gut immune environment. Immune cells of the same type are depicted in the same colour. Upward arrows correspond to increased activity, differentiation, generation or expression, whereas downward arrows indicate a decrease. Standard arrows represent cascades of events, with some arrows originating from metabolites and branching to different targets. An arrow pointing to another arrow denotes regulation of that pathway. Created in BioRender.

**FIGURE 2:**
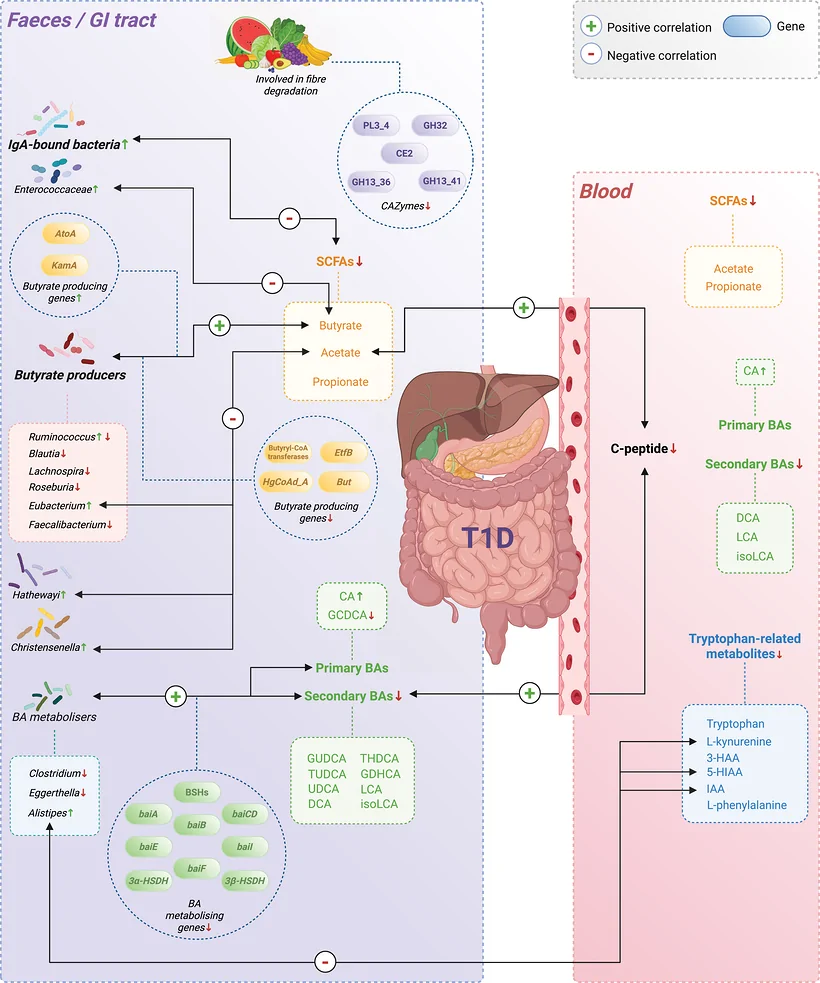
Schematic overview of current knowledge on T1D‐associated changes in gut microbial metabolism and biosynthesis. The purple panel illustrates observations derived from faecal sample analyses, representing GI tract status. In the pink panel, findings derived from plasma and serum analyses are categorised as blood. Arrows indicate positive (+) or negative (−) associations. Overall, decreased levels of various metabolites, including SCFAs (yellow), BAs (green) and tryptophan‐related metabolites (blue), have been observed, along with reduced pathway‐associated gene expression, as shown in their respective colours. Genes shown in purple represent CAZymes normally involved in fibre degradation. Alterations in the abundance of relevant gut microbes have been reported, although contradictory findings exist. Correlations between some of these bacteria and specific metabolites have been identified, as well as associations between certain metabolites and C‐peptide levels. Created in BioRender.

The gut microbiota can produce a vast array of metabolites, which interact with the host through receptor signalling, cellular uptake, enzyme inhibition or undergo hepatic metabolism. Over the years, most research has focussed on the effects of short‐chain fatty acids (SCFAs), secondary bile acids (BAs) and tryptophan‐derived metabolites (Figure 1). In T1D, changes in the abundance of these metabolites and associated microbes have been observed, as well as in the expression of genes associated with their metabolic pathways (Figure 2).

### 2.1 Short‐chain fatty acids

Dietary fibres, which are incompletely digested by host enzymes in the upper gastrointestinal (GI) tract, can be microbially fermented in the caecum and colon, producing SCFAs and various gasses and are a main energy source for colonocytes (Cummings et al., 1987). SCFAs are the most well‐known metabolites from this fermentation, and include acetate, propionate and butyrate. SCFA production is highly dependent on the types of gut bacteria present, which is influenced by host diet as well as environmental conditions and microbial cross‐feeding interactions (Mukhopadhya & Louis, 2025). SCFAs can exert their functions through interactions with G protein‐coupled receptors (GPCRs), including free fatty acid receptors (FFARs), which are widely expressed throughout the body, including on intestinal and immune cells (Priyadarshini et al., 2018).

Moreover, SCFAs can inhibit histone deacetylases (HDACs) (Stein & Riber, 2023) and, through their metabolism into acetyl‐CoA, can promote histone acetylation (Donohoe et al., 2012; Moffett et al., 2020), thereby increasing chromatin accessibility for gene transcription. HDAC inhibition further facilitates aryl hydrocarbon receptor (AhR) recruitment to its target gene promoter in the presence of tryptophan‐derived AhR agonists (Modoux et al., 2022). As a ligand‐dependent transcription factor, AhR orchestrates responses to environmental cues and plays a key role in various immunological and metabolic processes.

SCFAs are also known to promote intestinal integrity and homeostasis through various mechanisms, including prevention of pathogen colonisation (Mukhopadhya & Louis, 2025) and stimulation of mucin 2 production (Burger‐van Paassen et al., 2009).

Moreover, SCFA stimulation of innate immune cells results in decreased pro‐inflammatory cytokine production (tumour necrosis factor (TNF), interleukin (IL)‐1β and IL‐6), induction of a tolerant phenotype, and increased production of the anti‐inflammatory cytokine IL‐10) (Mukhopadhya & Louis, 2025). In adaptive immunity, SCFAs can induce regulatory T (Treg) cell generation and activation through HDAC inhibition (Arpaia et al., 2013). However, depending on their concentrations and the immunological milieu, SCFAs may contribute to a more inflammatory state. For instance, SCFAs can induce T cell differentiation into effector T helper (Th) 17 and Th1 cell subtypes during an active state of immunity, while in homeostatic conditions SCFAs increase IL‐10^+^ T cell proportions (Park et al., 2015). In a separate study, lower butyrate concentrations (0.1–0.25 mM) favoured Treg cell differentiation, while increased concentrations (0.5–1.0 mM) resulted in interferon (IFN)‐γ production by Treg and conventional T cells (Kespohl et al., 2017). Finally, SCFAs have been shown to enhance IgA‐mediated mucosal immunity (Kim et al., 2016) and induce B cell differentiation into IL‐10‐producing regulatory B (Breg) cells (Daïen et al., 2021).

SCFAs and their associated pathways have been increasingly studied in T1D. An early study in adults with T1D reported a reduced abundance of butyrate‐producing species, along with decreased expression of butyryl‐CoA transferase genes involved in butyrate production, and lower plasma levels of acetate and propionate (de Groot et al., 2017). Moreover, the T1D‐associated genus *Christensenella* was negatively correlated with faecal acetate levels. Similarly, faecal concentrations of acetate, butyrate and propionate were reduced in a study with paediatric T1D patients (Huang et al., 2020), with similar findings reported by Yuan et al. (2022). In support of a beneficial effect of SCFAs in T1D, faecal acetate levels were positively correlated with fasting C‐peptide, whereas the observed increased levels of IgA‐bound bacteria were negatively correlated with all three SCFAs (Huang et al., 2020). Additionally, faecal acetate and butyrate were found to be negatively correlated with *Eubacterium* and *Hathewayi*, and members of the *Enterococcaceae* family, respectively.

Compared with earlier studies, Yuan et al. (2022) identified additional butyrate‐producing genera (*Faecalibacterium*, *Blautia*, *Lachnospira*, *Ruminococcus* and *Roseburia*) and species (*Faecalibacterium prausnitzii*, *Eubacterium rectale* and *Roseburia intestinalis*) that were reduced in the gut microbiota of children with T1D compared to non‐diabetic subjects. Consistent with these findings, most genes involved in butyrate production (*EtfB* [electron transfer flavoprotein subunit β], *HgCoAd_A* [2‐hydroxyglutaryl‐CoA dehydratase subunit α] and *But* [butyryl‐CoA:acetate CoA‐transferase]) were decreased together with a significantly decreased abundance of carbohydrate‐active enzyme (CAZyme) genes, which are essential for complex carbohydrate breakdown. The longitudinal TEDDY study similarly reported higher levels of fermentation‐related gene and enrichment of SCFA biosynthesis pathways in control children compared with those with diabetes or islet autoimmunity (Vatanen et al., 2018). In line with reduced faecal butyrate, serum glucagon‐like peptide 1 (GLP‐1), whose secretion can be induced by butyrate, was also lowered (Yuan et al., 2022).

### 2.2 Secondary bile acids

Beyond their role in diet‐derived nutrient absorption, primary BAs exert systemic effects through BA signalling (Shapiro et al., 2018). In the intestine, gut microbes convert primary BAs into secondary BAs. Here, microbial bile salt hydrolases (BSHs) remove glycine and taurine from BAs via deconjugation, thereby reducing their reuptake by the enterohepatic circulation (Krautkramer et al., 2021). These unconjugated BAs subsequently undergo microbial modifications, including dehydroxylation, epimerisation and oxidation, resulting in the formation of secondary BAs. Secondary BAs may re‐enter the enterohepatic circulation and be reconjugated (Foley et al., 2019).

BA signalling is primarily mediated through interactions with the farnesoid X receptor (FXR), expressed in liver and intestine, and the Takeda G protein‐coupled receptor 5 (TGR5), which is predominantly expressed in the intestine (Fiorucci & Distrutti, 2015; Krautkramer et al., 2021). Primary BAs preferentially activate FXR, while secondary BAs predominantly engage TGR5 (Fiorucci & Distrutti, 2015).

Secondary BAs can enhance insulin sensitivity by facilitating GLP‐1 secretion from enteroendocrine cells (Katsuma et al., 2005) and by driving TGR5‐mediated differentiation of L cells (Lund et al., 2020). While certain pathogens may exploit secondary BA pathways to infect the host, these pathways and their metabolites can also restrict intestinal pathogen colonisation and growth (Foley et al., 2019). Likewise, secondary BAs can enhance intestinal integrity through upregulation of junctional proteins (Sun et al., 2021).

Secondary BAs also exhibit immunoregulatory effects. Deoxycholic acid (DCA) and lithocholic acid (LCA) suppress tumour necrosis factor (TNF)‐α production in bacterial antigen‐stimulated macrophages via GPBAR1 (Yoneno et al., 2013), while LCA further reduced inflammation in early necrotising enterocolitis through pregnane X receptor (PXR)‐mediated *Il6* and *Tlr4* (Toll‐like receptor 4) downregulation (Huang et al., 2018). In turn, 3‐oxolithocholic acid (3‐oxoLCA) inhibits Th17 cell differentiation by binding the transcription factor retinoid‐related orphan receptor‐γt (RORγt), while isoallolithocholic acid (isoalloLCA) promotes Treg cell differentiation through *Foxp3* (forkhead box P3) upregulation via mitochondrial reactive oxygen species (ROS) production (Hang et al., 2019). Likewise, a mixture of secondary BAs increased colonic retinoid‐related orphan receptor‐γ (RORγ)^+^ and FOXP3^+^ Treg cell in vivo, eventually contributing to reduced gut inflammation (Song et al., 2020).

In T1D, altered secondary BA metabolism has been linked to disease pathogenesis and progression. While children with new‐onset T1D exhibited reduced abundance of faecal BA‐metabolising genes and levels of the BAs glycoursodeoxycholic acid (GUDCA) and glycochenodeoxycholic acid (GCDCA), they showed increased levels of fibroblast growth factor 19 (FGF19), a negative regulator of hepatic BA synthesis (Yuan et al., 2022). Furthermore, a three‐year longitudinal study revealed that children, who developed multiple islet autoantibodies, exhibited distinct abundances of BA‐metabolising bacteria (*Alistipes*, *Clostridium*, *Eggerthella*, *Ruminococcus* and *Roseburia*) compared with those who developed a single autoantibody, with reduced *Clostridium* and *Eggerthella* and increased *Ruminococcus* at 18 and 24 months of age (Lamichhane et al., 2022). Prior to seroconversion, secondary BA biotransformation rates were significantly reduced, with decreased faecal levels of several taurine‐ and glycine‐conjugated secondary BAs (taurohyodeoxycholic acid [THDCA], tauroursodeoxycholic acid [TUDCA], ursodeoxycholic acid (UDCA), GUDCA and glycodeoxycholic acid [GDHCA]).

In adults with T1D, a recent study demonstrated significant reductions in faecal expression of BA‐induced (bai) operon genes (*baiB*, *baiCD* [nicotinamide adenine dinucleotide (NAD)^+^‐dependent 3‐oxo‐Δ^4^‐cholenoic acid oxidoreductase], *baiE* [bile acid 7α‐dehydratase], *baiF* [bile acid‐CoA hydrolase/bile acid‐CoA transferase] and *baiI* [bile acid 7β‐dehydratase/Δ^5^‐ketosteroid isomerase]) and hydroxysteroid dehydrogenase genes (*3α‐HSDH *[3α‐hydroxysteroid dehydrogenase] and *3β‐HSDH* [3β‐hydroxysteroid dehydrogenase]) (Liu et al., 2025). Correspondingly, faecal and serum profiles showed increased primary BA cholic acid (CA) alongside a reduction of several secondary BAs (DCA, LCA and isolithocholic acid [isoLCA]). In line with these changes, *Clostridium leptum* and *Clostridium scindens* abundances were significantly reduced. Notably, secondary BA levels positively correlated with fasting C‐peptide concentrations and C‐peptide area under the curve (AUC), linking BA metabolism with β‐cell function.

### 2.3 Tryptophan‐derived microbial metabolites

Tryptophan is an essential dietary amino acid that supports protein biosynthesis and serves as a precursor for a range of bioactive host‐ and microbiota‐derived metabolites (Agus et al., 2018; Xue et al., 2023). Tryptophan metabolism mainly occurs via the host kynurenine and serotonin pathway, and the bacterial indole pathway, and the lesser‐known interleukin 4‐induced gene 1 (IL4I1) cytokine‐induced pathway (Gabela et al., 2026). Gut microbes can influence both host pathways through their metabolites and expression of host enzymes (Agus et al., 2018; Gou et al., 2024; Zhao et al., 2025) or metabolise tryptophan into indole and its derivatives through activity of tryptophanase (TnA), tryptophan decarboxylase (TrD), tryptophan monooxygenase (TMO) and aromatic amino acid aminotransferase (ArAT) (Gou et al., 2024; Zhao et al., 2025). Several bacterial tryptophan‐derived catabolites can activate AhR, which may exert protective effects on both T1D pathogenesis and progression depending on the site, degree of activation and ligand type (Li et al., 2025).

In the intestine, AhR is expressed along a proximal‐to‐distal gradient in immune cells of the intestinal lamina propria and intestinal epithelial cells (IECs) (Lamas et al., 2018). In IECs, AhR signalling promotes epithelial cell renewal and turnover, and intestinal integrity. A mutual relationship exists between IECs and immune cells, whereby IEC ensure AhR ligand availability, while immune cells provide IL‐22, supporting IEC growth, survival and antimicrobial peptide production. Likewise, AhR expression by intraepithelial lymphocytes (IELs), innate lymphoid cells (ILCs), antigen presenting cells (APCs) and Th17/22 cells drives intestinal integrity and mucosal immune regulation.

Several microbial indole derivatives modulate intestinal immunity and barrier integrity through AhR signalling. For instance, indole‐3‐aldehyde induces AhR‐dependent IL‐22 production, resisting *Candida albicans* colonisation and mucosal inflammation (Zelante et al., 2013). Similarly, indoleacrylic acid induces AhR‐mediated IL‐10 production, upregulates goblet cell genes (*Muc2*, *St6gal1* [ST6 β‐galactoside α‐2,6‐sialyltransferase 1] and *C1galt1* [core 1 synthase, glycoprotein‐*N*‐acetylgalactosamine 3‐β‐galactosyltransferase 1]), and reduces IL‐6 and IL‐1β levels (Wlodarska et al., 2017). Furthermore, AhR activation by indole acetic acid (IAA) and indole propionic acid (IPA) reduces Th1 and Th17 cell abundance while increasing Treg and Th2 cell numbers and *Foxp3* expression, promoting an anti‐inflammatory response (Li et al., 2021).

Beyond AhR, indole and its derivatives can activate PXR (Zhao et al., 2025). IPA‐mediated PXR activation reduces intestinal inflammation and permeability, decreases enterocyte TNF‐α production and upregulates junctional protein‐coding mRNAs (*Ocln* [occludin], *Tjp1* [zonula occludens‐1 (ZO‐1)], *Cdh1* [E‐cadherin] and *Cldn7* [claudin‐7]) (Venkatesh et al., 2014).

In T1D, tryptophan metabolism plays a critical immunoregulatory role. Particularly, the activity of indoleamine 2,3‐dioxygenase 1 (IDO1), the rate‐limiting enzyme of the kynurenine pathway (Agus et al., 2018), is defective in T1D (Orabona et al., 2018). IDO1 catalyses the tryptophan‐to‐kynurenine conversion, limiting T cell proliferation and promoting Th1 cell apoptosis and Treg cell differentiation (Orabona et al., 2018). IDO1 expression is significantly decreased in insulin‐containing islets from double autoantibody‐positive donors and recent‐onset T1D patients, and is completely depleted in insulin‐deficient islets from T1D donors (Anquetil et al., 2018).

Alterations in tryptophan metabolite levels have been linked to T1D. However, available studies are limited, particularly regarding alterations in the indole pathway. Glutamic acid decarboxylase antibody‐positive adult T1D patients exhibited decreased serum levels of the tryptophan‐related metabolites 3‐hydroxyanthranilic acid, l‐kynurenine and l‐phenylalanine (Luo et al., 2022). Furthermore, the increased *Alistipes* abundance was negatively associated with l‐kynurenine, 5‐hydroxyindoleacetate and IAA. Similarly, serum tryptophan and kynurenine (Gürcü et al., 2020), as well as microbiota‐derived IAA (Zhang et al., 2022), were significantly lower in adults and children with T1D, respectively. Intriguingly, serum IAA was decreased in paediatric T1D slow progressors following seroconversion compared with rapid progressors (Lamichhane et al., 2023). Beyond tryptophan metabolism, several amino acid‐associated metabolic pathways, including phenylalanine, tyrosine and tryptophan biosynthesis, distinguished progression rates, potentially reflecting T1D‐associated dysregulations of saccharolytic fermentation (Vatanen et al., 2018), suggesting a shift towards increased microbial proteolytic activity as an alternative energy source in rapid progressors (Lamichhane et al., 2023).

## 3 GUT MICROBIOME‐BASED THERAPIES IN T1D

Gut microbiome‐based therapies in preclinical and clinical studies demonstrate encouraging results as potential intervention counteracting T1D.

### 3.1 Prebiotic interventions in T1D

Prebiotics are specific compounds utilised by microbiota that can beneficially influence host health or performance (Deehan et al., 2024). These benefits result from changes occurring in microbiota composition and/or activity due to direct or indirect effects from prebiotic usage.

In T1D murine models, prebiotics improve immunity, gut microbiota composition and disease severity, especially in prolonged treatment (Guimarães et al., 2024; Taylor & Vasu, 2021) (Table 1). Administration of yeast‐derived β‐glucan to non‐obese diabetic (NOD) mice increased gut mucosal expression of regulatory and Th17 cytokines (*Il10*, *Il17*, *Il12* and *Il21*), along with certain pro‐inflammatory cytokines (*Il1b* and *Ifng*) (Taylor & Vasu, 2021). Moreover, β‐glucan delayed hyperglycaemia, decreased insulitis severity and T1D incidence in the NOD mice.

**TABLE 1:** Preclinical outcomes of prebiotic interventions in T1D.

Prebiotic intervention	Study design	Clinical outcomes	Effects on gut microbiota	Reference
Yeast‐derived β‐glucan treatment vs. control	NOD mice Daily prebiotic solution (100 µL/100 µg) in 2‐week‐old mice for 7‐ or 30‐day treatment	**↓**T1D incidence **↓**Delayed hyperglycaemia **↓**Insulitis **↑**Colonic *Il10*, *Il17*, *Il12*, *Il21* expression **↑**Colonic *Ifng*, *Il1b* expression **↓**Colonic *Tnf* expression	**↓**α‐diversity **↑**β‐diversity **↓**Firmicutes, TM7, *Allobaculum*, *Oscillospira* **↑**Bacteroidetes, AF12, *Bacteroides*	Taylor & Vasu (2021)
Inulin supplementation vs. disease and healthy controls	STZ‐induction in 8‐ to 10‐week‐old mice 10% inulin was given 1 week before and 2 weeks after initial STZ dose	**↓**to 0% T1D incidence **↓**Blood glucose **↓**Insulitis **↑**Insulin **↑**Pancreatic IL‐10 **↑** *CLN* FOXP3, CCR4, *Ido1*, *Aldh1a2* expression **↑**Pancreatic Treg cells **↑** *CLN Ffar3*, Ffar2 and *Hcar2* expression **↑** *Colonic Muc1* expression, mucus production	**↑**Firmicutes, Verrucomicrobia, *Bifidobacterium*, *Clostridium* cluster IV, *Akkermansia muciniphila* **=** Faecal acetate, propionate **↑**Faecal butyrate	Guimarães et al. (2024)

In streptozotocin (STZ)‐induced diabetics, inulin supplementation fully protected against diabetes development and improved blood glucose pancreatic injury and insulin levels (Guimarães et al., 2024). Mechanistically, inulin restored pancreatic Treg cell abundance and IL‐10 production, while increasing FOXP3^+^ CCR4^+^ Treg cells in the caecal lymph nodes (CLNs). The insulin‐mediated effects appear to depend on Treg cell migration from CLNs to the pancreas, since genetic depletion of *Ccr4*, a key chemokine receptor, abolished the inulin‐mediated disease prevention and Treg cell infiltration of the pancreas. Inulin treatment also elevated faecal butyrate concentrations and gene expression of certain SCFA receptors.

Clinically, oligofructose‐enriched inulin supplementation in adolescents with T1D preserved C‐peptide levels, altered gut microbiota composition and showed a trend towards decreased intestinal permeability (Ho et al., 2019) (Table 2). Notably, a positive correlation between the lactulose‐to‐mannitol ratio and haemoglobin A1c (HbA1c) was observed, suggesting that improving intestinal permeability may enhance glycaemic control by decreasing systemic inflammation. This pilot study prompted a larger, ongoing multicentre trial (NCT04963777) in children and adults (*n* = 144) to investigate the impact of oligofructose‐enriched inulin on hypoglycaemia frequency and glycaemic control.

**TABLE 2:** Clinical outcomes of prebiotic interventions in T1D.

Prebiotic intervention	Study design	Clinical outcome	Effects on gut microbiota	Reference
Oligofructose‐enriched inulin supplementation vs. placebo Supplementation (8 g/day) for 12 weeks followed by 3‐month washout	Randomised, double‐blind, placebo‐controlled trial in adolescents (prebiotic *n* = 17; placebo *n* = 21) Mean age 12.52 ± 2.76 years, mean disease duration 7.31 ± 3.93 years (prebiotic group)	**↑**C‐peptide preservation **=** Serum HbA1c, GLP‐1, GLP‐2 **=** Serum IL‐6, IL‐10, TNF‐α, IFN‐γ	**↓** *Streptococcus*, *Roseburia inulinivorans*, *Terrisporobacter*, *Faecalitalea* **↑** *Bifidobacterium* associated with increased Coriobacteriales	Ho et al. (2019)

Altogether, prebiotic supplementation can affect T1D clinical outcomes, gut microbiota composition and intestinal permeability, while immune‐related effects are mainly restricted to preclinical models.

### 3.2 Probiotics as a supplementary therapy for T1D

Probiotics are live microorganisms, including bacteria and yeasts, with potential health benefits (Hill et al., 2014). Various factors influence probiotic efficacy, including baseline microbiota composition, host diet and administration route, resulting in variable outcomes across different studies.

Preclinical T1D research has predominantly addressed the application of *Akkermansia muciniphila* (Table 3). Given reports of decreased *Akkermansia* abundance in T1D, Hänninen et al. (2018) showed that *Akkermansia muciniphila* supplementation in NOD mice delayed T1D onset and promoted mucosal tolerance. This was associated with altered expression of macrophage pro‐ and anti‐inflammatory markers, enhanced mucus production and reduced expression of endoplasmic reticulum (ER)‐stress markers in IECs. Furthermore, islets and pancreatic lymph nodes (PLNs) exhibited an immunotolerogenic profile, with reduced TLR2 and TLR4 levels, increased *Il10* and *Tgfb* (transforming growth factor‐β) expression, elevated FOXP3^+^ Treg cells and reduced immune cell infiltration.

**TABLE 3:** Preclinical outcomes of probiotic therapy in T1D.

Probiotic intervention	Study design	Clinical outcomes	Effects on gut microbiota	Reference
*Akkermansia muciniphila* vs. disease control	NOD mice Thrice‐weekly probiotic (2 × 10^8^ CFU) administration from 3 to 10 weeks of age	**↓**Delay in T1D onset **↓**Pancreatic TLR2, TLR4 levels **↑**PLN *Il10*, *Tgfb* expression **↓**Pancreatic mononuclear leukocyte infiltration **↑**Pancreatic FOXP3^+^ Treg cells **↓**Colonic *Emr1*, *BiP* expression **↑**Colonic *Ym1*, *Reg3γ* expression **↑**Colonic mucus production **↓**Colonic crypt depth **↓**Serum endotoxin	**↓** *Ruminococcus torques*	Hänninen et al. (2018)
*Akkermansia muciniphila* vs. disease and healthy control	NOD mice. STZ‐induction in 8‐week‐old‐mice Probiotics (2 × 10^8^ CFU) every other day from day 0 to day 30 of STZ‐induction or thrice weekly from 8 to 13 weeks of age in NOD mice	**↓**T1D incidence **↓**Blood glucose **↑**Insulin **↓**Insulitis **↓**Serum anti‐insulin IgG **↑**Colonic IL‐22, IL‐10, TGF‐β, IFN‐γ levels **↑**Colonic *Ahr*, *Ffar2* expression **↑**Pancreatic and PLN FOXP3^+^ Treg cells, SIRP‐α^+^CD11b^+^CD103^+^ dendritic cells **↓**PLN Th17 cells, IgA^+^ B cells **=** Intestinal permeability	**↑**α‐diversity **↑**Firmicutes, Verrucomicrobiota, *Akkermansia muciniphila*, *Aerococcus*, *Acetatifactor*, *Corynebacterium*, *Lactobacillus*	Rodrigues et al. (2025)

CFU, colony‐forming units.

Rodrigues et al. (2025) further explored *Akkermansia muciniphila* supplementation in two T1D models (NOD and STZ‐induced diabetes), demonstrating reduced T1D incidence, improved blood glucose, preserved pancreatic islets, sustained serum insulin and reduced anti‐insulin antibodies. Supporting the role of gut microbiota in host signalling, colonic *Ahr* and colonic and pancreatic *Ffar2* expression were upregulated, along with colonic concentrations of several regulatory cytokines (IL‐22, IL‐10 and TGF‐β). The beneficial effects of *Akkermansia muciniphila* were additionally associated with elevated Treg and tolerogenic type‐2 dendritic cells and reduced Th17 and IgA^+^ B cells in the pancreas and PLNs.

Clinical T1D research has mainly focused on *Lactobacilli* and *Bifidobacteria* (Table 4). *Lactobacillus acidophilus* supplementation in children and adolescents with T1D improved several glycaemic markers, with decreased IL‐21 positively correlating with fasting blood glucose, HbA1c and total cholesterol, while increased IL‐22 showed negative correlations (Adly et al., 2025). However, effects on gut microbiota composition, additional glycaemic and inflammatory markers and long‐term clinical outcomes remain unexamined. In contrast, Groele et al. (2021) observed no effects on β‐cell function or immunity from combined *Lactobacillus rhamnosus* GG and *Bifidobacterium lactis* Bb12 supplementation in children, potentially due to a higher baseline thyroid autoimmunity in the probiotic group. Conversely, Mondanelli et al. (2020) found that *Lactobacillus rhamnosus* GG administration in children did improve the pro‐inflammatory profile, decreasing IFN‐γ and IL‐17F levels. Interestingly, circulating tryptophan increased while certain serotonin pathway metabolites decreased.

**TABLE 4:** Clinical outcomes of probiotic therapy in T1D.

Probiotic intervention	Study design	Clinical outcomes	Effects on gut microbiota	Reference
*Lactobacillus acidophilus* vs. placebo Daily supplementation (10^8^ CFU) for 6 months	Randomised, double‐blind, placebo‐controlled trial in children and adolescents (probiotic *n* = 33, placebo *n* = 34) Mean age 8.3 ± 2.6 years, mean disease duration 6.8 ± 3.3 years (probiotic group)	**↓**Fasting and random blood glucose **↓**HbA1c **↑**Serum IL‐22 **↓**Serum IL‐21	NA	Adly et al. (2025)
*Lactobacillus rhamnosus* GG and *Bifidobacterium lactis* Bb12 vs. placebo Daily supplementation (10^9^ CFU) for 6 months with follow‐up to 12 months	Randomised, double‐blind, placebo‐controlled trial in children (probiotic *n* = 43, placebo *n* = 44) Mean age 12.31 ± 2.13 years, mean disease duration 40.4 ± 16.2 days (probiotic group)	**=** C‐peptide AUC **=** HbA1c **=** Serum IL‐1β, IL‐2, IL‐10, TNF‐α, IFN‐γ **=** Serum zonulin	NA	Groele et al. (2021)
*Lactobacillus rhamnosus* GG vs. placebo Daily supplementation (10^9^/droplet) for 3 months	Randomised, placebo‐controlled trial in children (probiotic *n* = 36, placebo *n* = 25) Median age 14 [3–18] years (probiotic group)	**↓**PBMC‐derived IFN‐γ, IL‐17F **↑**Serum tryptophan **↓**Serum serotonin pathway metabolites **=** Serum kynurenine and indole pathway metabolites	NA	Mondanelli et al. (2020)
*Lactobacillus salivarius* subsp. *salicinius* AP‐32, *Lactobacillus johnsonii* MH‐68 and *Bifidobacterium animalis* subsp*. lactis* CP‐9 vs. placebo Daily supplementation (10^10^ CFU) for 6 months with 3‐month follow‐up	Randomised, double‐blind, placebo‐controlled trial in adolescents (probiotic *n* = 27, placebo *n* = 29) Mean age 14.1 ± 5.1 years, mean disease duration 74.4 ± 53.6 months (probiotic group)	**↓**Fasting blood glucose **↓**HbA1c **↑**Serum TGF‐β1 **↓**Serum IL‐8, TNF‐α, IL‐17, MIP‐1β, RANTES	**↑** *Bifidobacterium animalis*, *Lactobacillus salivarius*, *Akkermansia muciniphila*	Wang et al. (2022)
Vivomixx® (*Streptococcus thermophilus*, *Bifidobacterium infantis*, *Bifidobacterium longum*, *Bifidobacterium breve*, *Lactobacillus acidophilus*, *Lactobacillus plantarum*, *Lactobacillus paracasei*, *Lactobacillus delbrueckii* subsp*. bulgaricus*) vs. placebo Daily sachet (22.5 × 10^10^ CFU) for 6 months	Randomised, double‐blind, placebo‐controlled study in children (*n* = 27 probiotic, *n* = 23 placebo) Mean age 7.44 ± 3.42 years and median disease duration 40.5 (66) days (probiotic group)	**↓**Mean fasting and total blood glucose **↑**Median C‐peptide **↓**Median HbA1c **↓**Serum IA‐2 autoantibody **↑**Plasma IL‐10 **↑**PBMC‐derived induced Treg cells	NA	Lokesh et al. (2025)

CFU, colony‐forming units.

Beyond single‐strain and combined probiotic interventions, studies have investigated multi‐strain probiotics in T1D (Lokesh et al., 2025; Wang et al., 2022). Supplementation with *Lactobacillus salivarius *subsp.* salicinius *AP‐32,* Lactobacillus johnsonii* MH‐68 *and Bifidobacterium animalis *subsp.* lactis *CP‐9 altered gut microbiota composition and improved glycaemic and immune markers (Wang et al., 2022). Further analyses revealed correlations between the changed inflammatory markers and improvements in glycaemic outcomes. Notably, some of these effects persisted during follow‐up. Yet, the study did not elucidate individual strain contributions or determine whether the intervention affected pancreatic function.

A different placebo‐controlled randomised trial investigated the effects of the multi‐strain probiotic Vivomixx® in paediatric T1D patients (Lokesh et al., 2025). Supplementation of this bacterial consortium significantly reduced HbA1c and blood glucose levels, while increasing C‐peptide levels. Additionally, Vivomixx supplementation increased percentages of induced Treg cell, plasma IL‐10 and reduced insulin dose requirements and autoantibodies against insulinoma‐associated protein 2 (IA‐2).

Collectively, probiotic strain interventions appear to beneficially modulate glycaemic and inflammatory parameters in human T1D. Nevertheless, to date none of these interventions have resulted in disease remission or specifically tackle the autoimmune attack on β‐cells.

### 3.3 Postbiotics: Direct metabolite supplementation in T1D

Postbiotics contain non‐viable microbial cells and/or their byproducts, such as metabolites, cell fragments and proteins (Salminen et al., 2021). While the effects of probiotics depend on factors such as successful gut colonisation, viability in the GI tract, preparation, handling and safety, postbiotics are thought to circumvent these limitations and exert more direct effects on the host (Calvanese et al., 2025). In T1D, research has mainly focused on the use of SCFAs (Tables 5 and 6).

**TABLE 5:** Preclinical outcomes of postbiotic supplementation in T1D.

Postbiotic intervention	Study design	Clinical outcomes	Effects on gut microbiota	Reference
SCFA supplementation vs. disease control	NOD mice 8‐week‐old mice received two sodium butyrate injections (2 × 1 mg) for 1 week. Harvested islets were treated with various SCFAs including butyrate	**↓**T1D incidence **↑**Pancreatic CRAMP **↑**Pancreatic regulatory macrophages, ALDH^+^ dendritic cells, Treg cells **↓**Pancreatic IFN‐γ^+^CD8^+^ T cells	NA	Sun et al. (2015)
Sodium butyrate supplementation vs. disease control	STZ‐induction in mice Daily sodium butyrate administration (500 mg/kg) from day 0 of STZ‐induction for 6 weeks	**↓**Fasting blood glucose **↑**C‐peptide **↓**Islet injury **↑**Islet number, insulin‐positive islets **↓**HbA1c **↑**Pancreatic *Ins1*, *Ins2* expression **↓**Pancreatic *Il18*, *Tlr4* expression	NA	Yuan et al. (2022)
TUDCA supplementation vs. disease control	STZ‐induction in 8‐week‐old mice Following T1D onset, daily TUDCA injections (300 mg/kg) for 24 days	**↑**Glucose tolerance **↓**Fasting blood glucose **=** C‐peptide **↑**Insulin **↑**Pancreatic β‐cell mass, number **↓**Liver IDE activity	NA	Bronczek et al. (2019)
Comparison between HAMSA, HAMSB and HAMSAB diet vs. HAMS and control (NP) diet	NOD mice Mice were fed 15% acetylated and/or butyrylated HAMS for 5 weeks, starting at 5 or 10 weeks of age	**↓**to 0% T1D incidence*^HAMSAB^* **↓**Pancreatic immune cell infiltration*^HAMSAB^* **↑**Serum IL‐22*^HAMSA/^* ^H^ *^AMSB^* **↓**Serum IL‐21*^HAMSA^* **↑**PLN *Gata3*, *Gitr*, *Sell* expression*^HAMSB^* **↓**Splenic MHC‐I, CD86 expression*^HAMSA^* **↑**Colonic *Ocln* expression*^HAMSB^* **↓**Splenic, PLN and PP IGRP^+^ CD8^+^ cells, BDC2.5^+^ CD4^+^ cells, IgM^+^B220^+^ B cells*^HAMSA^* **↑**Splenic and PLN FOXP3^+^ CD4^+^ Treg cells*^HAMSB^*	**↓**Serum LPS*^HAMSA/HAMSB^*	Mariño et al. (2017)
HAMSAB vs. control diet	NOD mice 5‐week‐old mice were fed HAMSAB (15% acetylated and 15% butyrylated) for 5 weeks.	**↓**Disease incidence **↑**Pancreatic *Ins1*, *Iapp*, *G6pc2*, *Prlr*, *Ssr3* expression **↓**Pancreatic *Adm*‐*Ramp3* expression **↑**Pancreatic *JunB*, *Rel*, *Nfkb1*, *Malat1* expression **↓**Pancreatic *Fos*, *Impact*, *Stat1*, *Dusp1*, *Hsp40/Dnajb1* expression **↑**Pancreatic CD4^+^ T cells **↓**Pancreatic poly‐hormonal cells	NA	Vandenbempt et al. (2024)

**TABLE 6:** Clinical outcomes of postbiotic supplementation in T1D.

Postbiotic intervention	Study design	Clinical outcomes	Effects on gut microbiota	Reference
Sodium butyrate supplementation vs. placebo 4 g/day ingestion for 1 month, with 1‐month washout between treatments	Randomised, placebo‐controlled, double‐blind, crossover trial in adults (*n* = 30) Median age 32.5 [22‐61] years, mean disease duration 8 years	**=** Stimulated C‐peptide **=** Daily insulin dose **=** HbA1c **=** Innate, adaptive immunity **↓**PBMC‐derived IA‐2‐specific CD8^+^ T cells	**=** Gut microbiota **↓**Faecal propionate, butyrate	de Groot et al. (2020)
Sodium butyrate supplementation vs. placebo 3.6 g/day ingestion for 12 weeks.	Randomised, double‐blind, placebo‐controlled trial in adults with T1D, diabetic nephropathy and intestinal inflammation (postbiotic *n* = 28, *n* = 25 placebo) Mean age 54 ± 13 years, mean disease duration 30 ± 15 years	**=** HbA1c **=** Inflammatory markers **=** Metabolic profiles **=** Renal function, albuminuria **=** Intestinal inflammation	NA	Tougaard et al. (2022)
HAMSAB diet HAMSAB was consumed twice daily for 6 weeks (10 → 40 g/day), with 12‐week follow‐up	Single‐arm pilot study in adults (*n* = 18) Median age 36.5 [18‐45] years, mean disease duration 14 ± 12 years	**=** Mean daily average blood glucose **=** C‐peptide **=** Mean HbA1c **↑**Faecal proteomics: antioxidative capacity, intestinal integrity, anti‐inflammatory pathways **↓**Faecal *proteomics: c*ell‐death pathways **↑**PBMC‐derived CTLA4, TIGIT expression **↓**PBMC‐derived CD86 expression **↓**Plasma IL‐8, MIP‐1α, bFGF **↑**Serum tryptophan, kynurenine, xanthurenic acid	**↓**α‐diversity **↑** *Bacteroides uniformis*, unclassified *Parabacteroides*, *Parabacteroides distasonis* **↓** *Eubacterium ramulus*, *Eubacterium eligens*, *Coprococcus comes* **↑**Starch utilisation **↑**Faecal xanthurenic acid **↑**Plasma *and* faecal acetate, butyrate, propionate	Bell et al. (2022); Tillett et al. (2025)
HAMSAB vs. recommended diabetes diet Daily HAMSAB (10 gr + 1 gr/year of age) consumption for 4 weeks, with 4‐week washout between treatments	Crossover pilot study in youth (*n* = 7) Mean age 15 ± 1.2 years, mean disease duration 19.5 ± 6.3 years	**=** Blood glucose **=** C‐peptide AUC **=** HbA1c **↓**PBMC‐derived CCR6^+^, CD25^+^, BCL2^−^ GrB^+^, PD‐1^+^ MAIT cell	**↑** *Parabacteroides*, *Bifidobacterium* **=** Faecal acetate, propionate	Ismail et al. (2025)

In NOD mice, SCFAs can enhance β‐cell production of cathelicidin‐related antimicrobial peptide (CRAMP) via GPCR signalling (Sun et al., 2015). The resulting elevated CRAMP promotes a tolerogenic pancreatic environment, marked by higher frequencies of regulatory macrophages, aldehyde dehydrogenase (ALDH)^+^ dendritic cells and Treg cells, along with reduced IFN‐γ^+^ CD^+^ T cells, ultimately lowering T1D incidence.

Furthermore, oral butyrate supplementation in antibiotic‐treated mice receiving gut microbiota from paediatric T1D patients normalised fasting blood glucose and significantly improved insulin sensitivity (Yuan et al., 2022). Similarly, in STZ‐induced mice, butyrate reduced fasting blood glucose, glucose excursions and HbA1c, while increasing serum C‐peptide and demonstrating a trend towards higher serum insulin. It additionally reduced islet injury, increased islet number and insulin‐positive islets.

In contrast, 4‐week daily oral sodium butyrate supplementation in adults with T1D did not affect innate or adaptive immunity, glycaemic markers, nor β‐cell function (de Groot et al., 2020). Still, despite substantial baseline variation, a patient subset exhibited reduced autoreactive IA‐2‐specific CD8^+^ T cells. Interestingly, butyrate intake decreased faecal propionate and butyrate concentrations, and several microbial taxa discriminated treatment from placebo. Similarly, Tougaard et al. (2022) reported no inflammatory, glycaemic, metabolic or renal changes following 12‐week sodium butyrate treatment in adults with T1D, diabetic nephropathy and intestinal inflammation. Because both trials involved long‐standing T1D, it is plausible that the same intervention in new‐onset T1D patients with residual β‐cell function might yield different immunological and metabolic outcomes. Additionally, longer interventions and dose optimisation, both adapted to disease duration and severity, and larger sample sizes may also reveal more pronounced effects.

Alternatively, modified diets like high‐amylose maize‐resistant starch (HAMS) acetylated and butyrylated deliver high SCFA concentrations directly to the colon, as HAMS escapes small intestine digestion (Tables 5 and 6). In NOD mice, acetate‐ (HAMSA), butyrate‐ (HAMSB), or dual‐modified HAMS (HAMSAB) yielded distinct effects, with HAMSAB most effectively reducing islet infiltration and protecting against T1D (Mariño et al., 2017). Mechanistically, HAMSA reduced autoreactive T cell frequency in lymphoid tissues, potentially through altered B cell frequency, maturation and functionality (decreased major histocompatibility complex‐I (MHC‐I) and CD86 expression). Conversely, HAMSB increased Treg cell abundance and expression of functional markers *Gata3* (GATA‐binding protein 3), *Gitr* (glucocorticoid‐induced tumour necrosis factor receptor‐related protein) and *Sell* (selectin‐L). Both interventions improved intestinal integrity and inflammation, reducing serum lipopolysaccharide (LPS) and increasing IL‐22, with HAMSB enhancing *Ocln* expression and HAMSA lowering IL‐21.

Subsequent NOD mouse studies demonstrated that HAMSAB increased CD4^+^ T cell abundance and upregulated immunoregulatory genes (*JunB* [JunB proto‐oncogene], *Rel* [REL proto‐oncogene], *Nfkb1* [nuclear factor κ B subunit 1] and *Malat1* [metastasis associated lung adenocarcinoma transcript 1]) in pancreatic islets (Vandenbempt et al., 2024). It also reduced receptor‐ligand interactions between immune and endocrine cells (*Adm* [adrenomedullin] with *Ramp3* [receptor activity‐modifying protein 3]) and preserved endocrine identity, evidenced by fewer poly‐hormonal cells and lower expression of related markers. Furthermore, β‐cells upregulated key functional genes (*Ins1*, *Iapp* [islet amyloid polypeptide], *G6pc2* [glucose‐6‐phosphatase catalytic subunit 2], *Prlr* [prolactin receptor] and *Ssr3* [signal sequence receptor subunit 3]), while downregulating stress response genes (*Fos* [Fos proto‐oncogene], *Impact* [impact RWD domain protein], *Stat1* [signal transducer and activator of transcription 1], *Dusp1* [dual specificity phosphatase 1] and *Hsp40/Dnajb1* [DnaJ heat shock protein family (Hsp40) member B1]).

In human T1D, the HAMSAB diet has been investigated in adults and adolescents (Bell et al., 2022; Ismail et al., 2025). Although in adults, HAMSAB diet did not change glycaemic markers, plasma and stool SCFAs increased and B and T cells adopted more regulatory phenotypes, with a reduction in IL‐8, macrophage inflammatory protein‐1α (MIP‐1α), basic fibroblast growth factor (bFGF) and CD86 and an upregulation of the inhibitory markers cytotoxic T lymphocyte‐associated protein 4 (CTLA4) and T cell immunoreceptor with Ig and ITIM domains (TIGIT) (Bell et al., 2022). In adolescents, HAMSAB diet improved glucose excursions, but not postprandial C‐peptide, revealing an effect on glucose metabolism without affecting β‐cell secretory capacity (Ismail et al., 2025). Furthermore, HAMSAB diminished mucosal‐associated invariant T (MAIT) cell activation, evidenced by the reduction in MAIT cells positive for CCR6, CD25, B cell lymphoma 2 (BCL2)^−^ Granzyme B (GrB)^+^ and programmed cell death protein 1 (PD‐1)^+^. Lastly, HAMSAB partially affected composition and metabolic pathways of faecal microbiota. A follow‐up double‐blind, placebo‐controlled, phase 1B study (NCT06057454) with extended intervention duration to 12 weeks has recently been completed. Yet, results on adverse events, glucose and C‐peptide levels are still pending.

A follow‐up study in humanised gnotobiotic NOD mice showed that HAMSAB improved mucosal immunity, gut barrier integrity and metabolite production (Tillett et al., 2025). Indeed, faecal proteomics revealed enhanced antioxidative capacity and intestinal integrity, more anti‐inflammatory innate immune responses and reduced cell‐death associated pathways. HAMSAB also increased faecal and circulatory metabolites, including those in amino acid and nucleotide metabolic pathways such as tryptophan metabolism. Additionally, FMT from individuals with elevated faecal SCFAs into NOD mice downregulated intestinal expression of genes involved in tryptophan catabolism via the kynurenine pathway (*Kmo* [kynurenine 3‐monooxygenase] and *HAAO* [3‐hydroxyanthranilate 3,4‐dioxygenase]) and AhR ligand degradation (*Cyp1a1* [cytochrome P450 family 1 subfamily A member 1]). Consistently, FMT increased ileal AhR expression, and various glycaemic markers correlated with rates of plasma tryptophan metabolites, faecal AhR activity and SCFAs.

Besides SCFAs, the role of other microbial metabolites, such as secondary BA, is gaining attention in T1D. In STZ‐induced diabetes, the taurine‐conjugated form of the secondary BA UDCA, TUDCA, significantly reduced fasting blood glucose and improved glucose tolerance and plasma insulin (Bronczek et al., 2019), potentially through decreased hepatic insulin‐degrading enzyme (IDE) activity. TUDCA treatment additionally increased β‐cell mass and number per islet. Metabolic alterations were evident as well, with treated mice displaying increased glucose uptake and oxidation, whereas control mice relied predominantly on fatty acid oxidation. Building on these preclinical findings, a randomised, double‐blind, placebo‐controlled pilot study in adults with new‐onset T1D (*n* = 20) evaluated the effects of oral TUDCA, administered for 12 months, on β‐cell function (assessed by stimulated C‐peptide post‐MMT) (NCT02218619). Nonetheless, the trial did not meet its primary outcome (changes in β‐cell function) and remains unpublished to date.

Overall, SCFA‐based interventions favourably alter the immunoprofiling in patients with long‐standing T1D. However, they fail to improve β‐cell function, likely due to advanced disease stage. Other postbiotics, including different secondary BAs or tryptophan‐derived microbial metabolites, may also offer benefits and represent promising targets for future studies.

### 3.4 Synbiotics and combined therapies in T1D

Given the benefits of single bacterial strains or prebiotics in T1D, recent studies have focused on synbiotics and combination therapies. Synbiotics typically consist of a probiotic paired with a substrate (prebiotic) that can be utilised by the host's resident microorganisms or by the co‐administered probiotic (Swanson et al., 2020). In turn, combination therapies co‐administer probiotics with postbiotics.

To date, effects of a probiotic–postbiotic combination have only been explored preclinically in T1D (Table 7). In STZ‐treated rats, combining *Akkermansia muciniphila* and sodium butyrate greatly reduced T1D severity and improved glycaemia to near‐healthy‐control levels, outperforming individual treatments (Ghorbanian et al., 2025). Both the combination and the probiotic alone similarly increased hepatic SCFAs. However, it remains unclear whether this reflects changes in gut microbiota composition or activity.

**TABLE 7:** Preclinical outcomes of combined therapy in T1D.

Combined intervention	Study design	Clinical outcomes	Effects on gut microbiota	Reference
*Akkermansia muciniphila* and sodium butyrate supplementation vs. disease and healthy control	STZ‐induction in 8‐week‐old rats Following T1D onset, daily combination therapy (10^9^ CFU probiotic and 500 mg/kg sodium butyrate) was administered for 2 weeks	**↓**T1D severity **↓**Blood glucose **↑**Liver propionate, butyrate, acetate, valeric acid	NA	Ghorbanian et al. (2025)

Thus far, clinical studies in T1D are limited to synbiotics (Table 8). Zare Javid et al. (2020) demonstrated that supplementing *Lactobacillus sporogenes* GBI‐30 with maltodextrin and fructooligosaccharide improved glycaemic markers (HbA1c and insulin), oxidative stress and inflammatory markers (high‐sensitivity C‐reactive protein [hs‐CRP]). In contrast, a synbiotic formulation containing short‐chain fructooligosaccharides, *Lactobacillus reuteri*, *Lactobacillus rhamnosus* and *Bifidobacterium lactis* did not improve renal function, intestinal integrity or immunity in adult T1D patients with albuminuria (Stougaard et al., 2024). This lack of efficacy is likely attributable to advanced disease stage with comorbidities and short intervention.

**TABLE 8:** Clinical outcomes of synbiotic interventions in T1D.

Synbiotic intervention	Study design	Clinical outcomes	Effects on gut microbiota	Reference
*Lactobacillus sporogenes* GBI‐30, maltodextrin and fructooligosaccharide vs. placebo Daily supplementation (10^9^ CFU probiotic, 4:1 probiotic to prebiotic) for 8 weeks	Randomised, double‐blind, placebo‐controlled study in children (synbiotic *n* = 22, placebo *n* = 22) Mean age 10.36 ± 2.53 years, mean disease duration 4.45 ± 1.96 years (synbiotic group)	**↓**Mean HbA1c **↓**Mean hs‐CRP **↑**Mean insulin **↑**Mean total antioxidant capacity	NA	Zare Javid et al. (2020)
Short‐chain fructooligosaccharides, *Lactobacillus reuteri*, *Lactobacillus rhamnosus* and *Bifidobacterium lactis* vs. placebo Synbiotic mixture consumption (2856.0 mg prebiotic, 7.5 × 10^9^ CFU probiotic mix) twice daily for 12 weeks with 6‐week washout between treatments	Randomised, double‐blind, placebo‐controlled, crossover study in adults with albuminuria (*n* = 35) Mean age 58 ± 10 years, mean disease duration 35 ± 16 years	**=** UACR, PBR, GFR **=** Plasma TNF‐α, IL‐8, IL‐1β **↑**Plasma IL‐6 **=** Serum LPS, IFABP2	NA	Stougaard et al. (2024)

CFU, colony forming units.

### 3.5 Faecal microbiota transplantation in T1D

Among gut microbiome‐based therapies, FMT is the most investigated and implemented approach across a wide range of diseases. By transferring an entire microbial community and their associated metabolites, FMT may more drastically influence host gut microbiome composition and health (Kim et al., 2024). In T1D, FMT is still considered a relatively new approach, with only a few clinical trials conducted to date (Table 9).

**TABLE 9:** Clinical outcomes following FMT in T1D.

FMT intervention	Study design	Clinical outcomes	Effects on gut microbiota	Reference
Autologous vs. allogenic FMT FMT administration three times over 4 months, with follow‐up through 12 months	Randomised control trial in adults (autologous *n* = 10, allogenic *n* = 10) Mean age 25.0 ± 3.5 years, disease duration of <6 weeks (autologous group)	**↑**Mean fasting and stimulated C‐peptide preservation **↓**PBMC‐derived CD4^+^CXCR3^+^, CD8^+^CXCR3^+^ T cells **↑**Plasma 1‐arachidonoyl‐GPC, 1‐myristoyl‐2‐arachidonoyl‐GPC	**↑** *Desulfovibrio piger*	de Groot et al. (2021)
WMT treatment WMT administration over 3 consecutive days, with follow‐up through 3 months	Prospective, single‐centre study in adults with a discovery (T1D *n* = 44, control *n* = 34) and validation (T1D *n* = 21, control *n* = 12) cohort Mean age 32 ± 15 years, mean disease duration 5.5 ± 6.0 years (discovery)	**↑**Time‐in‐range **↑**C‐peptide AUC **↓**Daily insulin requirements **↓**HbA1c **↓**PBMC‐derived CD4^+^IL‐17A^+^ Th cells **↓**Serum IL‐17A, IL‐1β	**↑**α‐diversity, β‐diversity **↑**Faecal LCA, isoLCA **↓** *Escherichia coli*, *Streptococcus salivarius*, *Clostridium bolteae* **↑** *Faecalibacterium* sp., *Roseburia faecis*, *Dorea formicigenerans*, *Clostridium leptum*, *Firmicutes* sp. **↑**Faecal *baiE*, *BaiF*, *baiI* expression	Liu et al. (2025)
Autologous lyophilised faecal microbiota capsule treatment Daily intake for 3 months, with 3‐month run‐in and 3‐month follow‐up	Open‐label, single‐arm pilot study in adults (*n* = 10) Median age 24.0 [23.3‐30.5] years, median disease duration 2.0 [1.0‐3.0] years	**=** Stabilisation C‐peptide AUC, HbA1c, insulin requirement **↑**Duodenal *CCL22*, *CCL4*, *CD86*, *IFNG* expression **↑**Duodenal *CLDN12* expression	**=** α‐diversity **↑**β‐diversity	de Groen et al. (2025)

The first clinical study of FMT in recent‐onset T1D compared autologous with allogenic transplantation from healthy donors (de Groot et al., 2021). Autologous transplantation preserved mean fasting and stimulated C‐peptide, whereas the allogenic group deteriorated. While most T cell immunological changes were similar between groups, autologous treatment significantly decreased activated CD4^+^ and CD8^+^ T cell subsets expressing C‐X‐C motif chemokine receptor 3 (CXCR3), with changes in CD4^+^CXCR3^+^ T cells negatively correlating with C‐peptide AUC. Moreover, microbiome and metabolome analyses revealed that intestinal *Desulfovibrio piger* and *Prevotella* abundance correlated, respectively, positively and inversely with residual β‐cell function, whereas 1‐arachidonoyl‐*sn*‐glycero‐3‐phosphocholine and 1‐myristoyl‐2‐arachidonoyl‐*sn*‐glycero‐3‐phosphocholine positively correlating with fasting C‐peptide changes.

By contrast, Liu et al. (2025) studied washed microbiota transplantation (WMT), in T1D, focussing on secondary BA metabolism. WMT is an automated method of filtration and washing of donor stool to discard big particles, removing undigested residues, fungi, parasitic eggs and pro‐inflammatory metabolites, while concentrating beneficial bacteria (Zhang et al., 2022). WMT treatment in T1D patients significantly improved glycaemic control and reduced HbA1c rates, and significantly reduced IL‐17A, IL‐1β and Th17 cell frequency, while showing a trend towards increased Treg cell frequency. Furthermore, WMT restored gut microbial diversity, significantly decreasing pathogenic species (*Escherichia coli*, *Streptococcus salivarius* and *Clostridium bolteae*) and enriching SCFA producers (*Faecalibacterium* sp., *Roseburia faecis* and *Dorea formicigenerans*), alongside upregulating BA biosynthesis genes (*baiE*, *BaiF* and *baiI*). Although WMT did not affect stimulated C‐peptide in patients lacking baseline residual β‐cell function, responses varied in patients with residual β‐cell function. Subanalysis of responders showed distinct microbiome features, characterised by a marked increase in faecal secondary Bas, LCA and isoLCA. This suggests that FMT efficacy depends on baseline microbiota characteristics and residual β‐cell function, suggesting limited effectiveness in advanced disease.

Recently, Groen et al. demonstrated the safety and applicability of that encapsulated lyophilised FMT in patients with less than 4 years of T1D duration. In this single‐arm pilot study, autologous encapsulated FMT stabilised the initial decline in stimulated C‐peptide and increased expression of genes encoding for *CCL22*, *CCL4*, *CD86*, *IFNG* and *CLDN12* (claudin‐12). Moreover, β‐diversity of faecal microbiome shifted towards the capsule composition (de Groen et al., 2025). These promising results warrant further investigations with randomized placebo‐controlled trials.

## 4 CONCLUSION AND FUTURE DIRECTIONS

Various gut microbiome‐based therapies are currently being investigated in T1D, ranging from prebiotics to FMTs. These interventions exert to some degree beneficial effects, at least temporarily, on T1D pathogenesis and progression, although with variable results between studies. However, the field remains in its early stages, with most research conducted in preclinical or early‐phase clinical settings, lack of robust evidence and inconsistencies between studies. The strongest anti‐diabetic effects are primarily seen in preclinical models, although the majority of these positive findings are based on interventions prior to T1D onset. Therefore, there is a strong need for clinical trials to investigate the therapeutic potential of gut microbiome‐based interventions prior to disease onset (in seropositive or genetically at‐risk individuals) and at different stages of disease (e.g. new‐onset and long‐standing T1D), to elucidate their impact on both T1D prevention and disease progression. These interventions may represent adjuvant treatments to the current standard of care (insulin therapy) and to upcoming immunotherapies.

## AUTHOR CONTRIBUTIONS

S.P. Kok and E. Rampanelli: Conceptualisation, Writing—original draft. S.P. Kok, E. Rampanelli and M. Nieuwdorp: Writing—reviewing and editing. S.P. Kok: visualisation. E. Rampanelli and M. Nieuwdorp: supervision. All authors have read and approved the final version of the manuscript and agree to be accountable for all aspects of the work in ensuring that questions related to the accuracy or integrity of any part of the work are appropriately investigated and resolved. All persons designated as authors qualify for authorship, and all those who qualify for authorship are listed.

## CONFLICT OF INTEREST

M. Nieuwdorp is founder and a scientific advisory board member of *Caelus Pharmaceuticals* and *Advanced Microbiome Interventions (AMI)*, The Netherlands. He is also a board member of *Diabeter*. The paper reflects the views of the scientists and not the company. The other authors declare no conflicts of interest.

## GENERATIVE AI STATEMENT

All content was conceived, written and approved by the authors. Artificial intelligence (AI) tools were used to assist with figure preparation and for improving the readability of the review.

## References

[eph70434-bib-0001] Adly, A. A. M. , Ismail, E. A. R. , Abd‐Elgawad, M. M. , & Salah, N. Y. (2025). Probiotic supplementation improves glucose homeostasis and modulates interleukin (IL)‐21 and IL‐22 levels in pediatric patients with type 1 diabetes: A randomized placebo‐controlled trial. Metabolites, 15(5), 288. 40422866 10.3390/metabo15050288 PMC12113551

[eph70434-bib-0002] Agus, A. , Planchais, J. , & Sokol, H. (2018). Gut microbiota regulation of tryptophan metabolism in health and disease. Cell Host & Microbe, 23(6), 716–724. 29902437 10.1016/j.chom.2018.05.003

[eph70434-bib-0003] Anquetil, F. , Mondanelli, G. , Gonzalez, N. , Rodriguez Calvo, T. , Zapardiel Gonzalo, J. , Krogvold, L. , Dahl‐Jørgensen, K. , Van den Eynde, B. , Orabona, C. , Grohmann, U. , & von Herrath, M. G. (2018). Loss of IDO1 expression from human pancreatic β‐cells precedes their destruction during the development of type 1 diabetes. Diabetes, 67(9), 1858–1866. 29945890 10.2337/db17-1281 PMC6110313

[eph70434-bib-0004] Arpaia, N. , Campbell, C. , Fan, X. , Dikiy, S. , van der Veeken, J. , deRoos, P. , Liu, H. , Cross, J. R. , Pfeffer, K. , Coffer, P. J. , & Rudensky, A. Y. (2013). Metabolites produced by commensal bacteria promote peripheral regulatory T‐cell generation. Nature, 504(7480), 451–455. 24226773 10.1038/nature12726 PMC3869884

[eph70434-bib-0005] Bell, K. J. , Saad, S. , Tillett, B. J. , McGuire, H. M. , Bordbar, S. , Yap, Y. A. , Nguyen, L. T. , Wilkins, M. R. , Corley, S. , Brodie, S. , Duong, S. , Wright, C. J. , Twigg, S. , de St Groth, B. F. , Harrison, L. C. , Mackay, C. R. , Gurzov, E. N. , Hamilton‐Williams, E. E. , & Mariño, E. (2022). Metabolite‐based dietary supplementation in human type 1 diabetes is associated with microbiota and immune modulation. Microbiome, 10(1), 9. 35045871 10.1186/s40168-021-01193-9 PMC8772108

[eph70434-bib-0006] Blok, L. , Hanssen, N. , Nieuwdorp, M. , & Rampanelli, E. (2025). From microbes to metabolites: Advances in gut microbiome research in type 1 diabetes. Metabolites, 15(2), 138. 39997763 10.3390/metabo15020138 PMC11857261

[eph70434-bib-0007] Bronczek, G. A. , Vettorazzi, J. F. , Soares, G. M. , Kurauti, M. A. , Santos, C. , Bonfim, M. F. , Carneiro, E. M. , Balbo, S. L. , Boschero, A. C. , & Costa Júnior, J. M. (2019). The bile acid TUDCA improves beta‐cell mass and reduces insulin degradation in mice with early‐stage of type‐1 diabetes. Frontiers in Physiology, 10, 561. 31156453 10.3389/fphys.2019.00561 PMC6529580

[eph70434-bib-0008] Burger‐van Paassen, N. , Vincent, A. , Puiman, P. J. , van der Sluis, M. , Bouma, J. , Boehm, G. , van Goudoever, J. B. , van Seuningen, I. , & Renes, I. B. (2009). The regulation of intestinal mucin MUC2 expression by short‐chain fatty acids: Implications for epithelial protection. Biochemical Journal, 420(2), 211–219. 19228118 10.1042/BJ20082222

[eph70434-bib-0009] Calvanese, C. M. , Villani, F. , Ercolini, D. , & De Filippis, F. (2025). Postbiotics versus probiotics: Possible new allies for human health. Food Research International, 217, 116869. 40597563 10.1016/j.foodres.2025.116869

[eph70434-bib-0010] Cummings, J. H. , Pomare, E. W. , Branch, W. J. , Naylor, C. P. , & Macfarlane, G. T. (1987). Short chain fatty acids in human large intestine, portal, hepatic and venous blood. Gut, 28(10), 1221–1227. 3678950 10.1136/gut.28.10.1221 PMC1433442

[eph70434-bib-0011] Daïen, C. I. , Tan, J. , Audo, R. , Mielle, J. , Quek, L. E. , Krycer, J. R. , Angelatos, A. , Duraes, M. , Pinget, G. , Ni, D. , Robert, R. , Alam, M. J. , Amian, M. C. B. , Sierro, F. , Parmar, A. , Perkins, G. , Hoque, S. , Gosby, A. K. , Simpson, S. J. , … Macia, L. (2021). Gut‐derived acetate promotes B10 cells with antiinflammatory effects. Journal of Clinical Investigation Insight, 6(7), e144156. 33729999 10.1172/jci.insight.144156 PMC8119207

[eph70434-bib-0012] Deehan, E. C. , Al Antwan, S. , Witwer, R. S. , Guerra, P. , John, T. , & Monheit, L. (2024). Revisiting the concepts of prebiotic and prebiotic effect in light of scientific and regulatory progress—a consensus paper from the Global Prebiotic Association. Advances in Nutrition, 15(12), 100329. 39481540 10.1016/j.advnut.2024.100329 PMC11616045

[eph70434-bib-0013] de Groen, P. , Fuhri Snethlage, C. M. , Wortelboer, K. , Tokgöz, S. , Davids, M. , Verdoes, X. , Westerbeke, F. H. M. , Meijer, R. I. , Gotthardt, M. , de Vos, W. M. , Herrema, H. , Nieuwdorp, M. , & Hanssen, N. M. J. (2025). Autologous fecal microbiota capsules are safe and potentially preserve beta‐cell function in individuals with type 1 diabetes. Gut Microbes, 17(1), 2563155. 41047724 10.1080/19490976.2025.2563155 PMC12502823

[eph70434-bib-0014] de Groot, P. , Nikolic, T. , Pellegrini, S. , Sordi, V. , Imangaliyev, S. , Rampanelli, E. , Hanssen, N. , Attaye, I. , Bakker, G. , Duinkerken, G. , Joosten, A. , Prodan, A. , Levin, E. , Levels, H. , Potter van Loon, B. , van Bon, A. , Brouwer, C. , van Dam, S. , Simsek, S. , … Nieuwdorp, M. (2021). Faecal microbiota transplantation halts progression of human new‐onset type 1 diabetes in a randomised controlled trial. Gut, 70(1), 92–105. 33106354 10.1136/gutjnl-2020-322630 PMC7788262

[eph70434-bib-0015] de Groot, P. F. , Belzer, C. , Aydin, Ö. , Levin, E. , Levels, J. H. , Aalvink, S. , Boot, F. , Holleman, F. , van Raalte, D. H. , Scheithauer, T. P. , Simsek, S. , Schaap, F. G. , Olde Damink, S. W. M. , Roep, B. O. , Hoekstra, J. B. , de Vos, W. M. , & Nieuwdorp, M. (2017). Distinct fecal and oral microbiota composition in human type 1 diabetes, an observational study. PLOS ONE, 12(12), e0188475. 29211757 10.1371/journal.pone.0188475 PMC5718513

[eph70434-bib-0016] de Groot, P. F. , Nikolic, T. , Imangaliyev, S. , Bekkering, S. , Duinkerken, G. , Keij, F. M. , Herrema, H. , Winkelmeijer, M. , Kroon, J. , Levin, E. , Hutten, B. , Kemper, E. M. , Simsek, S. , Levels, J. H. M. , van Hoorn, F. A. , Bindraban, R. , Berkvens, A. , Dallinga‐Thie, G. M. , Davids, M. , … Nieuwdorp, M. (2020). Oral butyrate does not affect innate immunity and islet autoimmunity in individuals with longstanding type 1 diabetes: A randomised controlled trial. Diabetologia, 63(3), 597–610. 31915895 10.1007/s00125-019-05073-8

[eph70434-bib-0017] Donohoe, D. R. , Collins, L. B. , Wali, A. , Bigler, R. , Sun, W. , & Bultman, S. J. (2012). The Warburg effect dictates the mechanism of butyrate‐mediated histone acetylation and cell proliferation. Molecular Cell, 48(4), 612–626. 23063526 10.1016/j.molcel.2012.08.033 PMC3513569

[eph70434-bib-0018] Fiorucci, S. , & Distrutti, E. (2015). Bile acid‐activated receptors, intestinal microbiota, and the treatment of metabolic disorders. Trends in Molecular Medicine, 21(11), 702–714. 26481828 10.1016/j.molmed.2015.09.001

[eph70434-bib-0019] Foley, M. H. , O'Flaherty, S. , Barrangou, R. , & Theriot, C. M. (2019). Bile salt hydrolases: Gatekeepers of bile acid metabolism and host‐microbiome crosstalk in the gastrointestinal tract. PLOS Pathogens, 15(3), e1007581. 30845232 10.1371/journal.ppat.1007581 PMC6405046

[eph70434-bib-0020] Foster, N. C. , Beck, R. W. , Miller, K. M. , Clements, M. A. , Rickels, M. R. , DiMeglio, L. A. , Maahs, D. M. , Tamborlane, W. V. , Bergenstal, R. , Smith, E. , Olson, B. A. , & Garg, S. K. , & T1D Exchange Cinical Network . (2019). State of type 1 diabetes management and outcomes from the T1D Exchange in 2016–2018. Diabetes Technology & Therapeutics, 21(2), 66–72. 30657336 10.1089/dia.2018.0384 PMC7061293

[eph70434-bib-0021] Gabela, A. M. , Mthembu, N. , & Hadebe, S. (2026). Tryptophan metabolism in health and disease‐ implications for non‐communicable diseases. Immunology Letters, 277, 107093. 41015394 10.1016/j.imlet.2025.107093

[eph70434-bib-0022] Garcia‐Gutierrez, E. , O'Mahony, A. K. , Dos Santos, R. S. , Marroquí, L. , & Cotter, P. D. (2024). Gut microbial metabolic signatures in diabetes mellitus and potential preventive and therapeutic applications. Gut Microbes, 16(1), 2401654. 39420751 10.1080/19490976.2024.2401654 PMC11492678

[eph70434-bib-0023] Ghorbanian, F. , Seo, H. , Sarafraz, F. , Atashi, A. , Shuvo, M. S. H. , Chae‐eun, P. , Hossain, M. S. , Tajdozian, H. , Kim, S. , Song, H.‐Y. , & Yang, H. (2025). *In vivo* therapeutic potential of next‐generation probiotic *Akkermansia muciniphila* and butyrate combination therapy in diabetes. Journal of Microbiology and Biotechnology, 35(10), 1–12. 10.4014/jmb.2506.06025 PMC12603377 41162165

[eph70434-bib-0024] Gou, H. , Zeng, R. , Lau, H. C. H. , & Yu, J. (2024). Gut microbial metabolites: Shaping future diagnosis and treatment against gastrointestinal cancer. Pharmacological Research, 208, 107373. 39197712 10.1016/j.phrs.2024.107373

[eph70434-bib-0025] Groele, L. , Szajewska, H. , Szalecki, M. , Świderska, J. , Wysocka‐Mincewicz, M. , Ochocińska, A. , Stelmaszczyk‐Emmel, A. , Demkow, U. , & Szypowska, A. (2021). Lack of effect of *Lactobacillus rhamnosus GG* and *Bifidobacterium lactis Bb12* on beta‐cell function in children with newly diagnosed type 1 diabetes: A randomised controlled trial. British Medical Journal Open Diabetes Research & Care, 9(1), e001523. 10.1136/bmjdrc-2020-001523 PMC8006832 33771763

[eph70434-bib-0026] Guimarães, J. B. , Rodrigues, V. F. , Pereira, Í. S. , Manso, G. M. D. C. , Elias‐Oliveira, J. , Leite, J. A. , Waldetario, M. C. G. M. , de Oliveira, S. , Gomes, A. B. D. S. P. , Faria, A. M. C. , Ramos, S. G. , Bonato, V. L. D. , Silva, J. S. , Vinolo, M. A. R. , Sampaio, U. M. , Clerici, M. T. P. S. , & Carlos, D. (2024). Inulin prebiotic ameliorates type 1 diabetes dictating regulatory T cell homing via CCR4 to pancreatic islets and butyrogenic gut microbiota in murine model. Journal of Leukocyte Biology, 115(3), 483–496. 37947010 10.1093/jleuko/qiad132

[eph70434-bib-0027] Gürcü, S. , Girgin, G. , Yorulmaz, G. , Kılıçarslan, B. , Efe, B. , & Baydar, T. (2020). Neopterin and biopterin levels and tryptophan degradation in patients with diabetes. Scientific Reports, 10(1), 17025. 33046801 10.1038/s41598-020-74183-w PMC7552423

[eph70434-bib-0028] Hang, S. , Paik, D. , Yao, L. , Kim, E. , Trinath, J. , Lu, J. , Ha, S. , Nelson, B. N. , Kelly, S. P. , Wu, L. , Zheng, Y. , Longman, R. S. , Rastinejad, F. , Devlin, A. S. , Krout, M. R. , Fischbach, M. A. , Littman, D. R. , & Huh, J. R. (2019). Bile acid metabolites control TH17 and Treg cell differentiation. Nature, 576(7785), 143–148. 31776512 10.1038/s41586-019-1785-z PMC6949019

[eph70434-bib-0029] Hänninen, A. , Toivonen, R. , Pöysti, S. , Belzer, C. , Plovier, H. , Ouwerkerk, J. P. , Emani, R. , Cani, P. D. , & De Vos, W. M. (2018). *Akkermansia muciniphila* induces gut microbiota remodelling and controls islet autoimmunity in NOD mice. Gut, 67(8), 1445–1453. 29269438 10.1136/gutjnl-2017-314508

[eph70434-bib-0030] Heald, A. H. , Stedman, M. , Davies, M. , Livingston, M. , Alshames, R. , Lunt, M. , Rayman, G. , & Gadsby, R. (2020). Estimating life years lost to diabetes: Outcomes from analysis of National Diabetes Audit and Office of National Statistics data. Cardiovascular Endocrinology & Metabolism, 9(4), 183–185. 33225235 10.1097/XCE.0000000000000210 PMC7673790

[eph70434-bib-0031] Henriques, F. L. , Buckle, I. , & Forbes, J. M. (2025). Type 1 diabetes mellitus prevention: Present and future. Nature Reviews Endocrinology, 21(10), 608–622. 10.1038/s41574-025-01128-6 40527975

[eph70434-bib-0032] Hill, C. , Guarner, F. , Reid, G. , Gibson, G. R. , Merenstein, D. J. , Pot, B. , Morelli, L. , Canani, R. B. , Flint, H. J. , Salminen, S. , Calder, P. C. , & Sanders, M. E. (2014). The International Scientific Association for Probiotics and Prebiotics consensus statement on the scope and appropriate use of the term probiotic. Nature Reviews Gastroenterology & Hepatology, 11(8), 506–514. 24912386 10.1038/nrgastro.2014.66

[eph70434-bib-0033] Ho, J. , Nicolucci, A. C. , Virtanen, H. , Schick, A. , Meddings, J. , Reimer, R. A. , & Huang, C. (2019). Effect of prebiotic on microbiota, intestinal permeability, and glycemic control in children with type 1 diabetes. The Journal of Clinical Endocrinology & Metabolism, 104(10), 4427–4440. 31188437 10.1210/jc.2019-00481

[eph70434-bib-0034] Hou, K. , Wu, Z.‐X. , Chen, X.‐Y. , Wang, J.‐Q. , Zhang, D. , Xiao, C. , Zhu, D. , Koya, J. B. , Wei, L. , Li, J. , & Chen, Z.‐S. (2022). Microbiota in health and diseases. Signal Transduction and Targeted Therapy, 7(1), 135. 35461318 10.1038/s41392-022-00974-4 PMC9034083

[eph70434-bib-0035] Huang, C. , Rosolowsky, E. , Nour, M. A. , Butalia, S. , Ho, J. , Mayengbam, S. , Wang, W. , Pyke, S. , Virtanen, H. , & Reimer, R. A. (2025). Prebiotic supplementation in patients with type 1 diabetes: Study protocol for a randomised controlled trial in Canada. British Medical Journal Open, 15(5), e102486. 10.1136/bmjopen-2025-102486 PMC12128446 40449951

[eph70434-bib-0036] Huang, J. , Pearson, J. A. , Peng, J. , Hu, Y. , Sha, S. , Xing, Y. , Huang, G. , Li, X. , Hu, F. , Xie, Z. , Xiao, Y. , Luo, S. , Chao, C. , Wong, F. S. , Zhou, Z. , & Wen, L. (2020). Gut microbial metabolites alter IgA immunity in type 1 diabetes. Journal of Clinical Investigation Insight, 5(10), e135718. 32298241 10.1172/jci.insight.135718 PMC7259536

[eph70434-bib-0037] Huang, K. , Mukherjee, S. , DesMarais, V. , Albanese, J. M. , Rafti, E. , Draghi Ii, A. , Maher, L. A. , Khanna, K. M. , Mani, S. , & Matson, A. P. (2018). Targeting the PXR–TLR4 signaling pathway to reduce intestinal inflammation in an experimental model of necrotizing enterocolitis. Pediatric Research, 83(5), 1031–1040. 29360809 10.1038/pr.2018.14 PMC5959752

[eph70434-bib-0038] Huang, S. , Li, F. , Quan, C. , & Jin, D. (2024). Intestinal flora: A potential pathogenesis mechanism and treatment strategy for type 1 diabetes mellitus. Gut Microbes, 16(1), 2423024. 39520706 10.1080/19490976.2024.2423024 PMC11552262

[eph70434-bib-0039] International Diabetes Federation . (2025). IDF Diabetes Atlas (11th ed.). International Diabetes Federation. https://diabetesatlas.org/resources/idf‐diabetes‐atlas‐2025/

[eph70434-bib-0040] Ismail, H. M. , Liu, J. , Netherland, M. , Hasan, N. A. , Evans‐Molina, C. , & DiMeglio, L. A. (2025). Safety and effects of acetylated and butyrylated high‐amylose maize starch on youths recently diagnosed with type 1 diabetes: A pilot study. Diabetes, Obesity & Metabolism, 27(2), 987–992. 10.1111/dom.16039 PMC11700748 39467812

[eph70434-bib-0041] Katsuma, S. , Hirasawa, A. , & Tsujimoto, G. (2005). Bile acids promote glucagon‐like peptide‐1 secretion through TGR5 in a murine enteroendocrine cell line STC‐1. Biochemical and Biophysical Research Communications, 329(1), 386–390. 15721318 10.1016/j.bbrc.2005.01.139

[eph70434-bib-0042] Kespohl, M. , Vachharajani, N. , Luu, M. , Harb, H. , Pautz, S. , Wolff, S. , Sillner, N. , Walker, A. , Schmitt‐Kopplin, P. , Boettger, T. , Renz, H. , Offermanns, S. , Steinhoff, U. , & Visekruna, A. (2017). The microbial metabolite butyrate induces expression of Th1‐associated factors in CD4^+^ T cells. Frontiers in Immunology, 8, 1036. 28894447 10.3389/fimmu.2017.01036 PMC5581317

[eph70434-bib-0043] Kim, D.‐Y. , Lee, S.‐Y. , Lee, J.‐Y. , Whon, T. W. , Lee, J.‐Y. , Jeon, C. O. , & Bae, J.‐W. (2024). Gut microbiome therapy: Fecal microbiota transplantation vs live biotherapeutic products. Gut Microbes, 16(1), 2412376. 39377231 10.1080/19490976.2024.2412376 PMC11469438

[eph70434-bib-0044] Kim, M. , Qie, Y. , Park, J. , & Kim, C. H. (2016). Gut microbial metabolites fuel host antibody responses. Cell Host & Microbe, 20(2), 202–214. 27476413 10.1016/j.chom.2016.07.001 PMC4982788

[eph70434-bib-0045] Krautkramer, K. A. , Fan, J. , & Bäckhed, F. (2021). Gut microbial metabolites as multi‐kingdom intermediates. Nature Reviews Microbiology, 19(2), 77–94. 32968241 10.1038/s41579-020-0438-4

[eph70434-bib-0046] Lamas, B. , Natividad, J. M. , & Sokol, H. (2018). Aryl hydrocarbon receptor and intestinal immunity. Mucosal Immunology, 11(4), 1024–1038. 29626198 10.1038/s41385-018-0019-2

[eph70434-bib-0047] Lamichhane, S. , Sen, P. , Dickens, A. M. , Alves, M. A. , Härkönen, T. , Honkanen, J. , Vatanen, T. , Xavier, R. J. , Hyötyläinen, T. , Knip, M. , & Orešič, M. (2022). Dysregulation of secondary bile acid metabolism precedes islet autoimmunity and type 1 diabetes. Cell Reports Medicine, 3(10), 100762. 36195095 10.1016/j.xcrm.2022.100762 PMC9589006

[eph70434-bib-0048] Lamichhane, S. , Sen, P. , Dickens, A. M. , Kråkström, M. , Ilonen, J. , Lempainen, J. , Hyöty, H. , Lahesmaa, R. , Veijola, R. , Toppari, J. , Hyötyläinen, T. , Knip, M. , & Orešič, M. (2023). Circulating metabolic signatures of rapid and slow progression to type 1 diabetes in islet autoantibody‐positive children. Frontiers in Endocrinology, 14, 1211015. 37745723 10.3389/fendo.2023.1211015 PMC10516565

[eph70434-bib-0049] Li, H. , Fan, Y. , Liu, J. , Dong, S. , Wen, B. , Zhang, Y. , Wang, X. , Duan, X. , Hu, Y. , Yan, Z. , Shang, H. , & Jing, Y. (2025). Aryl hydrocarbon receptor in health and disease. MedComm, 6(11), e70426. 41112724 10.1002/mco2.70426 PMC12531456

[eph70434-bib-0050] Li, K. , Hao, Z. , Du, J. , Gao, Y. , Yang, S. , & Zhou, Y. (2021). *Bacteroides thetaiotaomicron* relieves colon inflammation by activating aryl hydrocarbon receptor and modulating CD4^+^T cell homeostasis. International Immunopharmacology, 90, 107183. 33229197 10.1016/j.intimp.2020.107183

[eph70434-bib-0051] Liu, Q. , Hua, Y. , He, R. , Xiang, L. , Li, S. , Zhang, Y. , Chen, R. , Qian, L. , Jiang, X. , Wang, C. , Li, Y. , Wu, H. , & Liu, Y. (2025). Restoration of intestinal secondary bile acid synthesis: A potential approach to improve pancreatic β cell function in type 1 diabetes. Cell Reports Medicine, 6(5), 102130. 40347938 10.1016/j.xcrm.2025.102130 PMC12147892

[eph70434-bib-0052] Lokesh, M. N. , Kumar, R. , Jacob, N. , Sachdeva, N. , Rawat, A. , Yadav, J. , & Dayal, D. (2025). Supplementation of high‐strength oral probiotics improves immune regulation and preserves beta cells among children with new‐onset type 1 diabetes mellitus: A randomised, double‐blind placebo control trial. Indian Journal of Pediatrics, 92(3), 277–283. 38557820 10.1007/s12098-024-05074-5

[eph70434-bib-0053] Lund, M. L. , Sorrentino, G. , Egerod, K. L. , Kroone, C. , Mortensen, B. , Knop, F. K. , Reimann, F. , Gribble, F. M. , Drucker, D. J. , de Koning, E. J. P. , Schoonjans, K. , Bäckhed, F. , Schwartz, T. W. , & Petersen, N. (2020). L‐cell differentiation is induced by bile acids through GPBAR1 and paracrine GLP‐1 and serotonin signaling. Diabetes, 69(4), 614–623. 32041793 10.2337/db19-0764 PMC7224989

[eph70434-bib-0054] Luo, S. , Yue, T. , Liu, Z. , Yang, D. , Xu, M. , Ding, Y. , Jiang, W. , Xu, W. , Yan, J. , Weng, J. , & Zheng, X. (2022). Gut microbiome and metabolic activity in type 1 diabetes: An analysis based on the presence of GADA. Frontiers in Endocrinology, 13, 938358. 36246882 10.3389/fendo.2022.938358 PMC9563112

[eph70434-bib-0055] Mariño, E. , Richards, J. L. , McLeod, K. H. , Stanley, D. , Yap, Y. A. , Knight, J. , McKenzie, C. , Kranich, J. , Oliveira, A. C. , Rossello, F. J. , Krishnamurthy, B. , Nefzger, C. M. , Macia, L. , Thorburn, A. , Baxter, A. G. , Morahan, G. , Wong, L. H. , Polo, J. M. , & Moore, R. J. , … Mackay, C. R. (2017). Gut microbial metabolites limit the frequency of autoimmune T cells and protect against type 1 diabetes. Nature Immunology, 18(5), 552–562. 28346408 10.1038/ni.3713

[eph70434-bib-0056] Modoux, M. , Rolhion, N. , Lefevre, J. H. , Oeuvray, C. , Nádvorník, P. , Illes, P. , Emond, P. , Parc, Y. , Mani, S. , Dvorak, Z. , & Sokol, H. (2022). Butyrate acts through HDAC inhibition to enhance aryl hydrocarbon receptor activation by gut microbiota‐derived ligands. Gut Microbes, 14(1), 2105637. 35895845 10.1080/19490976.2022.2105637 PMC9336500

[eph70434-bib-0057] Moffett, J. R. , Puthillathu, N. , Vengilote, R. , Jaworski, D. M. , & Namboodiri, A. M. (2020). Acetate revisited: A key biomolecule at the nexus of metabolism, epigenetics and oncogenesis—part 1: Acetyl‐CoA, acetogenesis and acyl‐CoA short‐chain synthetases. Frontiers in Physiology, 11, 580167. 33281616 10.3389/fphys.2020.580167 PMC7689297

[eph70434-bib-0058] Mondanelli, G. , Orecchini, E. , Volpi, C. , Panfili, E. , Belladonna, M. L. , Pallotta, M. T. , Moretti, S. , Galarini, R. , Esposito, S. , & Orabona, C. (2020). Effect of probiotic administration on serum tryptophan metabolites in pediatric type 1 diabetes patients. International Journal of Tryptophan Research, 13, 1178646920956646. 33061415 10.1177/1178646920956646 PMC7534075

[eph70434-bib-0059] Mukhopadhya, I. , & Louis, P. (2025). Gut microbiota‐derived short‐chain fatty acids and their role in human health and disease. Nature Reviews Microbiology, 23(10), 635–651. 40360779 10.1038/s41579-025-01183-w

[eph70434-bib-0061] Ogle, G. D. , Wang, F. , Haynes, A. , Gregory, G. A. , King, T. W. , Deng, K. , Dabelea, D. , James, S. , Jenkins, A. J. , Li, X. , Ma, R. C. W. , Maahs, D. M. , Oram, R. A. , Pihoker, C. , Svensson, J. , Zhou, Z. , Magliano, D. J. , & Maniam, J. (2025). Global type 1 diabetes prevalence, incidence, and mortality Estimates 2025: results from the international diabetes federation Atlas, 11th edition, and the t1d index version 3.0. Diabetes Research and Clinical Practice, 225, 112277. 40412624 10.1016/j.diabres.2025.112277

[eph70434-bib-0062] Orabona, C. , Mondanelli, G. , Puccetti, P. , & Grohmann, U. (2018). Immune checkpoint molecules, personalized immunotherapy, and autoimmune diabetes. Trends in Molecular Medicine, 24(11), 931–941. 30236470 10.1016/j.molmed.2018.08.005

[eph70434-bib-0063] Park, J. , Kim, M. , Kang, S. G. , Jannasch, A. H. , Cooper, B. , Patterson, J. , & Kim, C. H. (2015). Short‐chain fatty acids induce both effector and regulatory T cells by suppression of histone deacetylases and regulation of the mTOR–S6K pathway. Mucosal Immunology, 8(1), 80–93. 24917457 10.1038/mi.2014.44 PMC4263689

[eph70434-bib-0064] Pathak, V. , Pathak, N. M. , O'Neill, C. L. , Guduric‐Fuchs, J. , & Medina, R. J. (2019). Therapies for type 1 diabetes: Current scenario and future perspectives. Clinical Medicine Insights: Endocrinology and Diabetes, 12, 1179551419844521. 31105434 10.1177/1179551419844521 PMC6501476

[eph70434-bib-0065] Priyadarshini, M. , Kotlo, K. U. , Dudeja, P. K. , & Layden, B. T. (2018). Role of short chain fatty acid receptors in intestinal physiology and pathophysiology. Comprehensive Physiology, 8(3), 1091–1115. 29978895 10.1002/cphy.c170050 PMC6058973

[eph70434-bib-0066] Rawshani, A. , Sattar, N. , Franzén, S. , Rawshani, A. , Hattersley, A. T. , Svensson, A.‐M. , Eliasson, B. , & Gudbjörnsdottir, S. (2018). Excess mortality and cardiovascular disease in young adults with type 1 diabetes in relation to age at onset: A nationwide, register‐based cohort study. The Lancet, 392(10146), 477–486. 10.1016/S0140-6736(18)31506-X PMC6828554 30129464

[eph70434-bib-0067] Rodrigues, V. F. , Elias‐Oliveira, J. , Pereira, Í. S. , Pereira, J. A. , Barbosa, S. C. , Machado, M. S. G. , Guimarães, J. B. , Pacheco, T. C. F. , Bortolucci, J. , Zaramela, L. S. , Bonato, V. L. D. , Silva, J. S. , Martins, F. S. , Alves‐Filho, J. C. , Gardinassi, L. G. , Reginatto, V. , & Carlos, D. (2025). *Akkermansia muciniphila* restrains type 1 diabetes onset by eliciting cDC2 and Treg cell differentiation in NOD and STZ‐induced experimental models. Life Sciences, 372, 123624. 40204069 10.1016/j.lfs.2025.123624

[eph70434-bib-0068] Salminen, S. , Collado, M. C. , Endo, A. , Hill, C. , Lebeer, S. , Quigley, E. M. M. , Sanders, M. E. , Shamir, R. , Swann, J. R. , Szajewska, H. , & Vinderola, G. (2021). The International Scientific Association of Probiotics and Prebiotics (ISAPP) consensus statement on the definition and scope of postbiotics. Nature Reviews Gastroenterology & Hepatology, 18(9), 649–667. 33948025 10.1038/s41575-021-00440-6 PMC8387231

[eph70434-bib-0069] Shapiro, H. , Kolodziejczyk, A. A. , Halstuch, D. , & Elinav, E. (2018). Bile acids in glucose metabolism in health and disease. Journal of Experimental Medicine, 215(2), 383–396. 29339445 10.1084/jem.20171965 PMC5789421

[eph70434-bib-0070] Song, X. , Sun, X. , Oh, S. F. , Wu, M. , Zhang, Y. , Zheng, W. , Geva‐Zatorsky, N. , Jupp, R. , Mathis, D. , Benoist, C. , & Kasper, D. L. (2020). Microbial bile acid metabolites modulate gut RORγ^+^ regulatory T cell homeostasis. Nature, 577(7790), 410–415. 31875848 10.1038/s41586-019-1865-0 PMC7274525

[eph70434-bib-0071] Sorini, C. , Cosorich, I. , Lo Conte, M. , De Giorgi, L. , Facciotti, F. , Lucianò, R. , Rocchi, M. , Ferrarese, R. , Sanvito, F. , Canducci, F. , & Falcone, M. (2019). Loss of gut barrier integrity triggers activation of islet‐reactive T cells and autoimmune diabetes. Proceedings of the National Academy of Sciences, USA, 116(30), 15140–15149. 10.1073/pnas.1814558116 PMC6660755 31182588

[eph70434-bib-0072] Stein, R. A. , & Riber, L. (2023). Epigenetic effects of short‐chain fatty acids from the large intestine on host cells. microLife, 4, uqad032. 37441522 10.1093/femsml/uqad032 PMC10335734

[eph70434-bib-0073] Stewart, C. J. , Ajami, N. J. , O'Brien, J. L. , Hutchinson, D. S. , Smith, D. P. , Wong, M. C. , Ross, M. C. , Lloyd, R. E. , Doddapaneni, H. , Metcalf, G. A. , Muzny, D. , Gibbs, R. A. , Vatanen, T. , Huttenhower, C. , Xavier, R. J. , Rewers, M. , Hagopian, W. , Toppari, J. , Ziegler, A.‐G. , … Petrosino, J. F. (2018). Temporal development of the gut microbiome in early childhood from the TEDDY study. Nature, 562(7728), 583–588. 30356187 10.1038/s41586-018-0617-x PMC6415775

[eph70434-bib-0074] Stougaard, E. B. , Tougaard, N. H. , Sivalingam, S. , Hansen, C. S. , Størling, J. , Hansen, T. W. , Frimodt‐Møller, M. , Steinert, R. E. , Varasteh, S. , Groop, P.‐H. , Salmenkari, H. , Lehto, M. J. , Persson, F. , & Rossing, P. (2024). Effects of probiotics and fibers on markers of nephropathy, inflammation, intestinal barrier dysfunction and endothelial dysfunction in individuals with type 1 diabetes and albuminuria. The ProFOS Study. Journal of Diabetes and its Complications, 38(12), 108892. 39471704 10.1016/j.jdiacomp.2024.108892

[eph70434-bib-0075] Sun, J. , Furio, L. , Mecheri, R. , van der Does, A. M. , Lundeberg, E. , Saveanu, L. , Chen, Y. , van Endert, P. , Agerberth, B. , & Diana, J. (2015). Pancreatic β‐Cells limit autoimmune diabetes via an immunoregulatory antimicrobial peptide expressed under the influence of the gut microbiota. Immunity, 43(2), 304–317. 26253786 10.1016/j.immuni.2015.07.013

[eph70434-bib-0076] Sun, R. , Xu, C. , Feng, B. , Gao, X. , & Liu, Z. (2021). Critical roles of bile acids in regulating intestinal mucosal immune responses. Therapeutic Advances in Gastroenterology, 14, 17562848211018098. 34104213 10.1177/17562848211018098 PMC8165529

[eph70434-bib-0077] Swanson, K. S. , Gibson, G. R. , Hutkins, R. , Reimer, R. A. , Reid, G. , Verbeke, K. , Scott, K. P. , Holscher, H. D. , Azad, M. B. , Delzenne, N. M. , & Sanders, M. E. (2020). The International Scientific Association for Probiotics and Prebiotics (ISAPP) consensus statement on the definition and scope of synbiotics. Nature Reviews Gastroenterology & Hepatology, 17(11), 687–701. 32826966 10.1038/s41575-020-0344-2 PMC7581511

[eph70434-bib-0078] Taylor, H. B. , & Vasu, C. (2021). Impact of prebiotic β‐glucan treatment at juvenile age on the gut microbiota composition and the eventual type 1 diabetes onset in non‐obese diabetic mice. Frontiers in Nutrition, 8, 769341. 34805251 10.3389/fnut.2021.769341 PMC8595985

[eph70434-bib-0079] Tillett, B. J. , Dwiyanto, J. , Secombe, K. R. , George, T. , Zhang, V. , Anderson, D. , Duggan, E. , Giri, R. , Loo, D. , Stoll, T. , Morrison, M. , Begun, J. , Hill, M. M. , Gurzov, E. N. , Bell, K. J. , Saad, S. , Barlow, C. K. , Creek, D. J. , Chong, C. W. , … Hamilton‐Williams, E. E. (2025). SCFA biotherapy delays diabetes in humanized gnotobiotic mice by remodeling mucosal homeostasis and metabolome. Nature Communications, 16(1), 2893. 10.1038/s41467-025-58319-y PMC11937418 40133336

[eph70434-bib-0080] Tougaard, N. H. , Frimodt‐Møller, M. , Salmenkari, H. , Stougaard, E. B. , Zawadzki, A. D. , Mattila, I. M. , Hansen, T. W. , Legido‐Quigley, C. , Hörkkö, S. , Forsblom, C. , Groop, P.‐H. , Lehto, M. , & Rossing, P. (2022). Effects of butyrate supplementation on inflammation and kidney parameters in type 1 diabetes: A randomized, double‐blind, placebo‐controlled trial. Journal of Clinical Medicine, 11(13), 3573. 35806857 10.3390/jcm11133573 PMC9267418

[eph70434-bib-0081] Vandenbempt, V. , Eski, S. E. , Brahma, M. K. , Li, A. , Negueruela, J. , Bruggeman, Y. , Demine, S. , Xiao, P. , Cardozo, A. K. , Baeyens, N. , Martelotto, L. G. , Singh, S. P. , Mariño, E. , Gysemans, C. , & Gurzov, E. N. (2024). HAMSAB diet ameliorates dysfunctional signaling in pancreatic islets in autoimmune diabetes. iScience, 27(1), 108694. 38213620 10.1016/j.isci.2023.108694 PMC10783594

[eph70434-bib-0082] Vatanen, T. , Franzosa, E. A. , Schwager, R. , Tripathi, S. , Arthur, T. D. , Vehik, K. , Lernmark, Å. , Hagopian, W. A. , Rewers, M. J. , She, J.‐X. , Toppari, J. , Ziegler, A.‐G. , Akolkar, B. , Krischer, J. P. , Stewart, C. J. , Ajami, N. J. , Petrosino, J. F. , Gevers, D. , Lähdesmäki, H. , … Xavier, R. J. (2018). The human gut microbiome in early‐onset type 1 diabetes from the TEDDY study. Nature, 562(7728), 589–594. 30356183 10.1038/s41586-018-0620-2 PMC6296767

[eph70434-bib-0083] Venkatesh, M. , Mukherjee, S. , Wang, H. , Li, H. , Sun, K. , Benechet, A. P. , Qiu, Z. , Maher, L. , Redinbo, M. R. , Phillips, R. S. , Fleet, J. C. , Kortagere, S. , Mukherjee, P. , Fasano, A. , Le Ven, J. , Nicholson, J. K. , Dumas, M. E. , Khanna, K. M. , & Mani, S. (2014). Symbiotic bacterial metabolites regulate gastrointestinal barrier function via the xenobiotic sensor PXR and Toll‐like Receptor 4. Immunity, 41(2), 296–310. 25065623 10.1016/j.immuni.2014.06.014 PMC4142105

[eph70434-bib-0084] Wang, C.‐H. , Yen, H.‐R. , Lu, W.‐L. , Ho, H.‐H. , Lin, W.‐Y. , Kuo, Y.‐W. , Huang, Y.‐Y. , Tsai, S.‐Y. , & Lin, H.‐C. (2022). Adjuvant probiotics of *Lactobacillus salivarius* subsp. *salicinius AP‐32*, *L. johnsonii MH‐68*, and *Bifidobacterium animalis* subsp. *lactis CP‐9* attenuate glycemic levels and inflammatory cytokines in patients with type 1 diabetes mellitus. Frontiers in Endocrinology, 13, 754401. 35299968 10.3389/fendo.2022.754401 PMC8921459

[eph70434-bib-0085] Wlodarska, M. , Luo, C. , Kolde, R. , d'Hennezel, E. , Annand, J. W. , Heim, C. E. , Krastel, P. , Schmitt, E. K. , Omar, A. S. , Creasey, E. A. , Garner, A. L. , Mohammadi, S. , O'Connell, D. J. , Abubucker, S. , Arthur, T. D. , Franzosa, E. A. , Huttenhower, C. , Murphy, L. O. , Haiser, H. J. , … Xavier, R. J. (2017). Indoleacrylic acid produced by commensal *Peptostreptococcus* species suppresses inflammation. Cell Host & Microbe, 22(1), 25–37.e6.e26. 28704649 10.1016/j.chom.2017.06.007 PMC5672633

[eph70434-bib-0086] Xue, C. , Li, G. , Zheng, Q. , Gu, X. , Shi, Q. , Su, Y. , Chu, Q. , Yuan, X. , Bao, Z. , Lu, J. , & Li, L. (2023). Tryptophan metabolism in health and disease. Cell Metabolism, 35(8), 1304–1326. 37352864 10.1016/j.cmet.2023.06.004

[eph70434-bib-0087] Yoneno, K. , Hisamatsu, T. , Shimamura, K. , Kamada, N. , Ichikawa, R. , Kitazume, M. T. , Mori, M. , Uo, M. , Namikawa, Y. , Matsuoka, K. , Sato, T. , Koganei, K. , Sugita, A. , Kanai, T. , & Hibi, T. (2013). TGR5 signalling inhibits the production of pro‐inflammatory cytokines by in vitro differentiated inflammatory and intestinal macrophages in Crohn's disease. Immunology, 139(1), 19–29. 23566200 10.1111/imm.12045 PMC3634536

[eph70434-bib-0088] Yuan, X. , Wang, R. , Han, B. , Sun, C. , Chen, R. , Wei, H. , Chen, L. , Du, H. , Li, G. , Yang, Y. U. , Chen, X. , Cui, L. , Xu, Z. , Fu, J. , Wu, J. , Gu, W. , Chen, Z. , Fang, X. , Yang, H. , … Luo, F. (2022). Functional and metabolic alterations of gut microbiota in children with new‐onset type 1 diabetes. Nature Communications, 13(1), 6356. 10.1038/s41467-022-33656-4 PMC9606127 36289225

[eph70434-bib-0089] Zare Javid, A. , Aminzadeh, M. , Haghighi‐zadeh, M. H. , & Jamalvandi, M. (2020). The effects of synbiotic supplementation on glycemic status, lipid profile, and biomarkers of oxidative stress in type 1 diabetic patients. A placebo‐controlled, double‐blind, randomized clinical trial. Diabetes, Metabolic Syndrome and Obesity, 13, 607–617. 10.2147/DMSO.S238867 PMC7060036 32184640

[eph70434-bib-0090] Zelante, T. , Iannitti, R. G. , Cunha, C. , De Luca, A. , Giovannini, G. , Pieraccini, G. , Zecchi, R. , D'Angelo, C. , Massi‐Benedetti, C. , Fallarino, F. , Carvalho, A. , Puccetti, P. , & Romani, L. (2013). Tryptophan catabolites from microbiota engage aryl hydrocarbon receptor and balance mucosal reactivity via interleukin‐22. Immunity, 39(2), 372–385. 23973224 10.1016/j.immuni.2013.08.003

[eph70434-bib-0091] Zhang, J. , Wu, W. , Huang, K. , Dong, G. , Chen, X. , Xu, C. , Ni, Y. , & Fu, J. (2022). Untargeted metabolomics reveals gender‐ and age‐ independent metabolic changes of type 1 diabetes in Chinese children. Frontiers in Endocrinology, 13, 1037289. 36619558 10.3389/fendo.2022.1037289 PMC9813493

[eph70434-bib-0092] Zhao, P. , Chen, Y. , Zhou, S. , & Li, F. (2025). Microbial modulation of tryptophan metabolism links gut microbiota to disease and its treatment. Pharmacological Research, 219, 107896. 40763909 10.1016/j.phrs.2025.107896

